# Decoding the stoichiometric composition and organisation of bacterial metabolosomes

**DOI:** 10.1038/s41467-020-15888-4

**Published:** 2020-04-24

**Authors:** Mengru Yang, Deborah M. Simpson, Nicolas Wenner, Philip Brownridge, Victoria M. Harman, Jay C. D. Hinton, Robert J. Beynon, Lu-Ning Liu

**Affiliations:** 1 Institute of Integrative Biology, University of Liverpool, Crown Street, Liverpool, L69 7ZB UK; 2 College of Marine Life Sciences, and Frontiers Science Center for Deep Ocean Multispheres and Earth System, Ocean University of China, 266003 Qingdao, China

**Keywords:** Proteomics, Mass spectrometry, Organelles, Bacterial pathogenesis

## Abstract

Some enteric bacteria including *Salmonella* have evolved the propanediol-utilising microcompartment (Pdu MCP), a specialised proteinaceous organelle that is essential for 1,2-propanediol degradation and enteric pathogenesis. Pdu MCPs are a family of bacterial microcompartments that are self-assembled from hundreds of proteins within the bacterial cytosol. Here, we seek a comprehensive understanding of the stoichiometric composition and organisation of Pdu MCPs. We obtain accurate stoichiometry of shell proteins and internal enzymes of the natural Pdu MCP by QconCAT-driven quantitative mass spectrometry. Genetic deletion of the major shell protein and absolute quantification reveal the stoichiometric and structural remodelling of metabolically functional Pdu MCPs. Decoding the precise protein stoichiometry allows us to develop an organisational model of the Pdu metabolosome. The structural insights into the Pdu MCP are critical for both delineating the general principles underlying bacterial organelle formation, structural robustness and function, and repurposing natural microcompartments using synthetic biology for biotechnological applications.

## Abstract (web summary)

Enteric pathogens such as *Salmonella* depend on propanediol-utilising microcompartments (Pdu MCP), which self-assemble from cytosolic proteins. Using mass spectrometry-based absolute quantification, the authors here define the protein stoichiometry and propose an organizational model of a *Salmonella* Pdu MCP.

## Introduction

Self-assembly of proteins into various complexes ranging from the nanometre to the micrometre scale is ubiquitous, and the resulting systems play pivotal roles in many biological processes^1^. The process of self-assembly inherently defines the supramolecular structures of protein complexes, confers structural stability, and could include a regulatory dimension.

The bacterial microcompartment (BMC) is a paradigm of self-assembling macromolecular complexes and is a subcellular organelle assembled from many thousands of protein subunits of 10–20 distinct types^2^. The BMC is composed of a single-layer protein shell that resembles an icosahedral viral capsid, with facets composed of hexameric and trimeric proteins and vertices capped by pentameric proteins, and internal enzymes encapsulated by the shell^3–7^. The shell proteins possess a central pore that is key to modulating passage of metabolites and thus facilitating the catalytic activities of internal enzymes^3,4,8–10^. In addition, segmenting and compartmentalising key enzymatic pathways inside the BMC permit enhanced metabolic performance and protect the cell from toxic or volatile intermediates. These organisational features allow BMCs to play critical roles in a range of biological processes, including CO_2_ fixation, pathogenesis, and microbial ecology, across a broad range of bacterial species^11–13^.

**Fig. 1:**
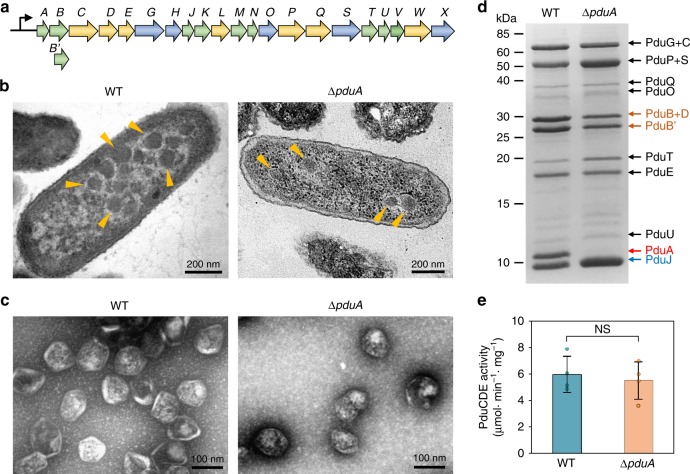
The Pdu microcompartments and their isolation from *S*. Typhimurium LT2. **a** Diagram of the *pdu* operon, which encodes structural genes (*pduABB’JKMNTU*) and catalytic genes (*pduCDELPQW* for 1,2-PD degradation and *pduGHOSX* for B_12_ recycling) of the Pdu MCP. **b** Thin-section transmission electron microscopy of the WT and Δ*pduA* cells showing the presence of Pdu MCPs (yellow arrows). **c** Negative staining TEM images of purified Pdu MCPs from the WT and Δ*pduA* cells, demonstrating the structural integrity of isolated Pdu MCPs. **d** SDS-PAGE of purified Pdu MCPs from the WT and Δ*pduA* cells, revealing the polypeptide composition. PduA was detected in the WT Pdu MCPs but was absent in the Δ*pduA* Pdu MCPs (red arrow), confirming the genetic deletion of *pduA*. PduJ possesses an elevated abundance in the Δ*pduA* Pdu MCPs compared to the WT form (blue arrow), whereas the content of PduB, PduB′, and PduD in the ΔpduA Pdu MCPs were reduced (yellow arrows). **e** PduCDE (diol dehydratase) activity of purified Pdu MCPs from the WT and Δ*pduA* cells, demonstrating the functional integrity of isolated Pdu MCPs. The WT and Δ*pduA* Pdu MCPs had similar propanediol dehydratase (PduCDE) activity normalised by the total protein amount of the samples (*p* = 0.651, *n* = 4, two-sided *t* test). The centre for error bars represents the mean, and error bars represent SD. *n* number of biologically independent experiments. Source data of Fig. 1e is provided as a Source Data file.

The anabolic carboxysome, the CO_2_-fixing organelle in cyanobacteria and some chemoautotrophs, was the first BMC to be discovered^14^. Other catabolic BMCs, or metabolosomes, have evolved for utilisation of diverse carbon sources, for example, the 1,2-propanediol (1,2-PD)-utilising microcompartment (Pdu MCP) in *Salmonella* and other enteric bacteria, which plays vital roles in 1,2-PD degradation and enteric pathogenesis^9,15–17^. In *Salmonella*, the genes related to Pdu MCP formation and function are located in a single *pdu* operon that encodes a total of 22 proteins (Fig. 1a). The shell of the Pdu MCP contains nine types of proteins: PduA, B, B’, M, N, J, K, T, and U^16^. PduA, B, B’, and J are the major shell components, whereas PduK, M, N, T, and U have relatively low abundance in the Pdu MCP shell^18^. Among the shell proteins, PduN forms pentamers occupying the vertices of the polyhedral structure^16,19^. The Pdu MCP shell encapsulates enzymes and cofactors for 1,2-PD degradation^13^ and confines propionaldehyde, a product of 1,2-PD degradation, to protect the bacterial cell from toxic sequelae^20^. The catalytic enzymes of 1,2-PD degradation pathway consist of propanediol dehydratase (PduCDE), PduL, PduP, PduQ, and PduW. PduCDE (diol dehydratase) is the major enzyme in the organelle to catalyse the conversion of 1,2-PD to propionaldehyde^21^. Propionaldehyde is then converted to propionyl coenzyme A (propionyl-CoA) or 1-propanol by PduP (aldehyde dehydrogenase) or PduQ (alcohol dehydrogenase), respectively^22,23^. PduL catalyses the conversion of propionyl-CoA to propionyl-PO_4_^2- ^^24^, and PduW converts the resulting propionyl-PO_4_^2-^ to propionate, transferring the phosphate group to ADP to generate ATP^25^. In addition, the 1,2-PD metabolism requires the enzymes for the reactivation of diol dehydratase and vitamin B_12_ recycling, including PduS (cobalamin reductase)^26^, PduO (adenosyltransferase)^27^, PduGH (diol dehydratase reactivase)^15^, and PduX (L-threonine kinase)^28^. PduV, a protein of unknown function, was proposed to connect with the cytoskeleton and be responsible for positioning of Pdu MCPs in the bacterial cytoplasm^29^.

The fundamental nature of self-assembly has provoked great interest in the adoption of these principles for bioinspired design and the engineering of functional protein self-assemblies for biotechnological applications. However, we still have limited knowledge about the biogenesis and self-assembly of BMCs; particularly, the exact stoichiometric composition and organisation of BMCs, as the key features of the self-assembly, have not been established. To address these questions requires precise quantification approach as well as preparation and analysis of functional BMCs.

Here we perform absolute quantification of protein components in the active Pdu MCPs from *Salmonella enterica* serovar Typhimurium (*S*. Typhimurium) LT2, by exploiting quantitative mass spectrometry using a QconCAT (concatamer of standard peptides for absolute quantification) standard^30,31^. We define the stoichiometry of individual shell proteins and interiors within the Pdu MCP. The encapsulation of Pdu enzymes within the Pdu MCP is characterised by live-cell confocal microscopy and electron microscopy (EM). The accurate protein stoichiometry allows us to propose a structural model of the Pdu MCP. Our findings provide insights into the organising principles of Pdu MCPs, which could be extended to other BMCs and self-assembling macromolecular complexes, and may inform strategies for the bespoke engineering of functional metabolosomes in biotechnology applications.

## Results

### Formation and isolation of functional Pdu MCPs

The genes involved in 1,2-PD metabolism form a continuous cluster on the *Salmonella* chromosome, at the *pdu* locus, beginning with the *pduA* gene that encodes the canonical BMC shell protein PduA (Fig. 1a). The *pdu* operon is transcribed from a single transcriptional start site located at 25 nucleotides upstream of *pduA*^32,33^. To investigate the structural variability of distinct Pdu MCP forms and to verify the protein quantification strategy, we studied both the wild type (WT) *S*. Typhimurium LT2 and the LT2-Δ*pduA* deletion mutant (Supplementary Fig. 1, Supplementary Tables 1 and 2). The increased growth rate of the LT2-Δ*pduA* strain compared with the WT in the presence of 1,2-PD at limiting levels of vitamin B_12_ (Supplementary Fig. 2) probably reflects the altered organisation of Pdu MCP particles and the semi-permeability of a remodelled shell that allows access of substrates to internal enzymes, as reported previously^16,34^. The formation of Pdu MCPs in the WT and Δ*pduA* cells grown in the microcompartment-inducing media (MIM) (see “Methods”) was confirmed by thin-section EM (Fig. 1b, arrows). In both strains, the Pdu MCP structures were polygonal with straight edges and angular facets, typical features of BMCs^35^.

The Pdu MCPs were isolated from the WT and the *pduA*-null mutant by differential centrifugation^36^. EM observations confirmed the polyhedral architecture of isolated Pdu MCPs, with the diameter ranging from 90 to 130 nm (Fig. 1c). The mean diameter of the Δ*pduA* Pdu MCP (102 ± 16 nm, *n* = 87) was slightly smaller than that of the WT forms (110 ± 13 nm, *n* = 104) (*p* = 1.12e^−4^, Supplementary Fig. 3). The mean thickness of the protein shell was 4.5 ± 0.6 nm (*n* = 156) for the WT and 4.3 ± 0.6 nm (*n* = 141) for the Δ*pduA* Pdu MCP (Supplementary Fig. 3). The presence of major Pdu proteins in the purified MCPs was indicated by sodium dodecyl sulfate–polyacrylamide gel electrophoresis (SDS-PAGE; Fig. 1d, Supplementary Fig. 4). PduA was detected in the WT Pdu MCPs but was absent from the Δ*pduA* Pdu MCP (Fig. 1d, red), confirming the genetic deletion of *pduA*. The minor Pdu MCP proteins (PduH, K, L, M, N, V, W, and X) were not visualised by SDS-PAGE, due to their low abundance within the Pdu MCP^36^. It was evident that the level of PduJ, one of the major shell proteins, was elevated in the Δ*pduA* Pdu MCP compared to the WT Pdu MCP (Fig. 1d, blue), whereas the content of PduB, PduB’, and PduD were reduced in the Δ*pduA* Pdu MCP (Fig. 1d, yellow). The isolated Pdu MCPs were enzymatically functional (Fig. 1e); the WT and Δ*pduA* Pdu MCPs had similar PduCDE activity normalised by the total protein amount of the samples (*p* = 0.651, *n* = 4, two-sided *t* test). Taken together, our results demonstrate the structural and functional integrity of the isolated Pdu MCPs; comparison of WT and Δ*pduA* Pdu MCPs suggests that the abundance of individual proteins and the overall structure of the functional Pdu MCP can be modulated.

### Absolute quantification of proteins within the Pdu MCP

**Fig. 2:**
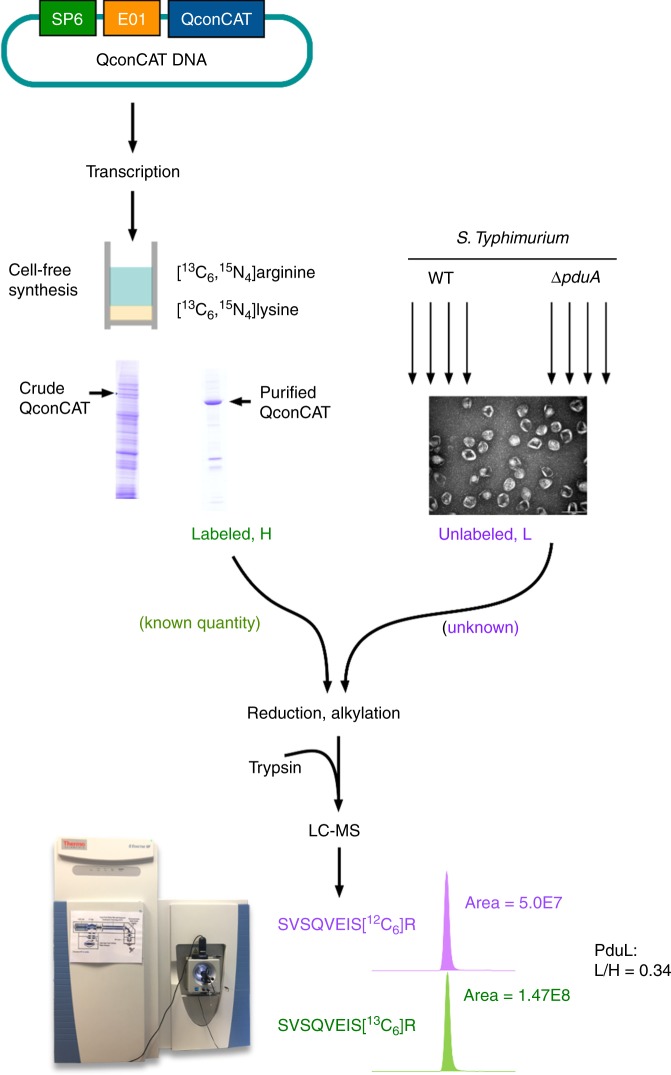
Schematic overview of QconCAT strategy. The QconCAT gene was subcloned into a plasmid vector (pEU01) optimised for cell-free system (CFS). The CFS was employed for transcription and translation to express the QconCAT proteins labelled with stable isotopes ([^13^C_6_,^15^N_4_]arginine and [^13^C_6_,^15^N_2_]lysine). The labelled QconCAT proteins (heavy, purple) were purified, quantified, and added to isolated Pdu MCPs from WT or Δ*pduA* cells with four replicated (unlabelled, light, green) in known quantity. After trypsin digestion, analyte mix released each of the QconCAT peptides in a strict stoichiometry of 1:1. LC-MS analysis allowed the quantification of each represented peptide of the isolated Pdu MCPs. One signature peptide for PduL, SVSQVEIS, was shown as an example.

Mass spectrometry has been used for the relative quantitation of Pdu MCP components but could not define the number of proteins per metabolosome^18^. To establish the accurate stoichiometry of all Pdu proteins within the isolated Pdu MCP, we used high-resolution liquid chromatography mass spectrometry (LC-MS) calibrated with protein-specific stable-isotope labelled internal standards generated via the QconCAT strategy (Fig. 2, Supplementary Note 1)^31,37^. The QconCAT approach is based on an artificial protein, a concatenation of multiple standard peptides that are quantotypic for the targeted proteins (Supplementary Table 3)^31^. A single, stable isotope-labelled QconCAT protein was produced by cell-free synthesis^30^, validated by SDS-PAGE (Supplementary Fig. 5). The QconCAT was detectable as a strong band at the expected mobility, clearly visible above the cell-free system (CFS) background. This QconCAT protein was spiked into the Pdu MCP samples and co-digested to generate a set of reference peptides for all Pdu MCP proteins that permitted absolute quantification of multiple proteins (Supplementary Fig. 6).

Because the Pdu MCPs were so highly purified and the Pdu MCP proteins are the most abundant in the samples (Supplementary Fig. 4), we elected to use an MS1 precursor QconCAT quantification. The purity of the Pdu MCP complexes was also evident from the base peak chromatograms of purified complexes compared with the complex chromatograms derived from lysed cell extracts (Supplementary Fig. 7). By contrast, the purified complex yield base peak intensity chromatograms that exhibited few peaks, each of which was baseline resolved from adjacent peaks, are exactly as would be anticipated from highly purified proteins, and indeed were confirmed by SDS-PAGE analysis of the Pdu MCPs (Supplementary Fig. 8a). Each precursor ion was cleanly isolated using the high-resolution and high-scanning speed of the MS1 approach. Further, replicate samples of lysed cell extracts and purified MCPs were analysed by label-free quantification (Supplementary Fig. 8b, c). Proteins were ranked according to abundance, as measured by label-free quantification, and the abundances of the shell and core proteins of the MCPs were highlighted on the ranked protein abundance curves. The combined label-free abundance of Pdu proteins is 96% of the total proteins in the MCP fraction compared with 15% in the whole-cell lysate. The Pdu MCP complexes were abundant in lysed cell extracts and were highly enriched after purification, lending weight to the utility of an MS1 approach.

The purified Pdu MCP proteins were mixed with QconCAT, co-digested, and analysed by high-resolution mass spectrometry of their precursor ions. This approach yielded a high number of sampling points across the chromatographic peak, and processing the data through Skyline^38^ permitted accurate integration of peak area. The representative extracted ion chromatograms for two peptides attest to the quality of the data and the ease with which the precursor ions could be recovered (Supplementary Fig. 9).

**Fig. 3:**
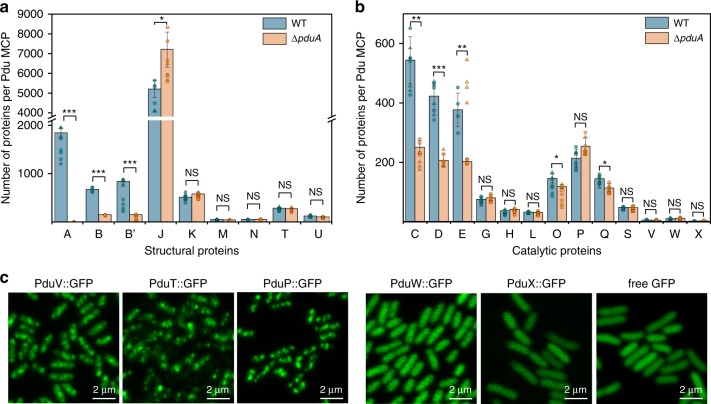
Stoichiometry and localisation of Pdu proteins. **a** Gross number of the structural proteins per Pdu MCP. **b** Gross number of the catalytic proteins per Pdu MCP. Circle: signature peptide 1; triangle: signature peptide 2. The peptides with higher values were used for generating error bars (SD) and centre (mean), reasoning that signal loss from endogenous peptide is more likely. One peptide with higher values for PduE was excluded, because a time course digest revealed that slower digestion of this peptide could have led to overestimation. The stoichiometric data are shown in Table 1. The deletion of PduA resulted in significant changes in the abundance of shell proteins PduB, B’, and PduJ (*p* = 7.110e^−7^, 2.022e^−8^, and 0.013, respectively, *n* = 4, two-sided *t* test) and interiors PduC, D, E, O, and Q (*p* = 0.001, 0.0003, 0.002, 0.027, and 0.028, respectively, *n* = 4, two-sided *t* test). *n* number of biologically independent samples. ns, *p* > 0.05; *p < 0.05; **p < 0.01; ***p < 0.001. **c** The subcellular distribution of PduV, PduW, and PduX in *S*. Typhimurium LT2. PduV, PduW, PduT, and PduP were fused with eGFP at their C-termini and recombined into the *S*. Typhimurium LT2 chromosome. The eGFP fused to the C-terminal of PduX and the native eGFP were plasmid encoded. The spotty distribution of PduV, similar to that observed for PduT and PduP, indicated that PduV is localised with the Pdu MCPs. In contrast, PduW and PduX were localised throughout the cytoplasm, comparable to native eGFP, and did not possess typical Pdu MCP distribution in vivo. Source data of Fig. 3a, b are provided as Source Data files.

We designed the QconCAT to deploy two unique peptides to quantify each Pdu MCP protein (Supplementary Tables 3 and 4). The agreement between the two peptides was excellent, yielding accurate quantification of all the proteins (except for PduE) (Fig. 3a, b, Supplementary Fig. 10). Digestion time-course analysis (Supplementary Fig. 11) revealed that one of the target peptides from PduE demonstrated differing digestion kinetics between the target protein and QconCAT standard. This peptide was then excluded. Table 1 presents the percentage of each Pdu protein within MCPs isolated from four biological replicates of WT and Δ*pduA S*. Typhimurium cells. Replicates of the same strain (WT vs WT, Δ*pduA* vs Δ*pduA*) were mostly consistent, while the PduA and PduB content differed between the WT and Δ*pduA* Pdu MCPs (Supplementary Fig. 12). A perfect icosahedral Pdu MCP will have 20 facets and contain 12 PduN pentamers at the vertices of the icosahedron^4,39^. Based on the value of 60 PduN molecules per Pdu MCP, we quantified the other Pdu proteins within a single Pdu MCP (Table 1).

**Table 1:** Absolute quantification of Pdu proteins in *S*. Typhimurium LT2-WT and LT2-Δ*pduA*.

**Category**	**Protein**		**Structure**	**% Total protein WT/Δ*pduA***	***n*** **WT/Δ*pduA***	***n***_**m**_ **WT/Δ*pduA***
Structural proteins	PduA	Hexamer^63^		15.8/0.1	1841 ± 102/10 ± 3	307 ± 17/2 ± 0
	PduB	Trimer^64^		5.8/1.6	671 ± 42/156 ± 7	224 ± 14/52 ± 2
	PduB′	Trimer^64^		7.1/1.6	834 ± 52/158 ± 13	278 ± 17/52 ± 4
	PduJ	Hexamer^44^		44.7/72.2	5212 ± 430/7202 ± 889	869 ± 72/1200 ± 148
	PduK	Hexamer?^a^		4.4/5.8	514 ± 41/580 ± 41	86 ± 7/97 ± 7
	PduM	?^b^		0.5/0.5	56 ± 9/50 ± 7	
	PduN	Pentamer^19^		0.5/0.6	60 ± 4/60 ± 8	12 ± 1/12 ± 2
	PduT	Trimer^63^		2.5/2.8	289 ± 19/276 ± 27	96 ± 6/92 ± 9
	PduU	Hexamer^10^		1.1/1.1	130 ± 17/111 ± 10	22 ± 3/18 ± 2
	Total			82.3/86.2	9607 ± 450/8602 ± 890	1893 ± 78/1525 ± 149
Catalytic proteins	PduC			4.7/2.5	543 ± 79/251 ± 22	
	PduD			3.6/2.1	423 ± 46/207 ± 23	
	PduE			2.4/2.8	376 ± 57/204 ± 8	
	PduG			0.6/0.8	76 ± 9/81 ± 10	
	PduH			0.3/0.4	38 ± 4/43 ± 4	
	PduL			0.3/0.3	33 ± 3/32 ± 5	
	PduO			1.2/1.2	146 ± 15/118 ± 7	
	PduP			1.8/2.6	214 ± 31/255 ± 29	
	PduQ			1.2/1.1	145 ± 12/114 ± 14	
	PduS			0.4/0.5	49 ± 4/48 ± 6	
	PduV			0.1/0.1	7 ± 2/6 ± 2	
	PduW			0.1/0.1	11 ± 2/13 ± 3	
	PduX			0.0/0.0	4 ± 1/4 ± 2	
	Total			17.7/13.8	2065 ± 115/1376 ± 48	

Source data are provided as a Source Data file.

*n* gross number of protein monomers per Pdu MCP, *n*_m_ number of protein multimers per Pdu MCP.

^a^PduK is presumed to be a hexamer because it has an ~70 amino acid extension on the C-terminus with low complexity that is probably disordered^63^.

^b^PduM is listed as a shell component according to ref. ^36^.

Of the structural components for the WT Pdu MCPs, PduJ was the most abundant hexameric shell protein (5212 copies of PduJ monomer per MCP), followed by the hexameric PduA (1841 monomers per Pdu MCP), trimeric PduB’ (834 monomers per Pdu MCP), and trimeric PduB (671 monomers per Pdu MCP) (Table 1). The ratio of distinct shell protein subunits (A:B:B’:J:K:M:N:T:U) was 31:11:14:87:9:1:1:5:2 (Table 2). PduJ accounted for 54.2% of all shell proteins by number, higher than the sum of PduA, PduB, and PduB’ (34.8%), demonstrating the key role of PduJ in tiling the shell. Apart from the major shell proteins, we also quantified the abundance of minor Pdu shell proteins (PduK, M, N, T, and U) (Table 1). There were 514 copies of PduK monomers per Pdu MCP. The number of trimeric PduT (289 monomers per Pdu MCP) was more than twice that of hexameric PduU (130 monomers per Pdu MCP), which is different from the PduT/PduU ratio of 1:2 estimated by two-dimensional electrophoresis^18^. PduM has been reported to be a Pdu MCP structural protein^36^, and our data revealed 56 copies of PduM subunits per Pdu MCP, comparable with the content of less abundant shell pentamer PduN (normalised as 60 monomers per Pdu MCP).

**Table 2:** Different ratios calculated from the data of gross number of proteins per Pdu MCP.

**Ratio of gross contents of Pdu proteins**	**WT**	**Δ*pduA***	**Reported values for WT**^b^
A:B:B’:J:K:M:N:T:U^a^	31:11:14:87:9:1:1:5:2	0:3:3:120:10:1:1:5:2	10:6:7:15:1:—:—:1:2^18^
PduC:PduD:PduE	1:1:1	1:1:1	1:1:1^39^; 2:1:2^18^
PduG:PduH	2:1	2:1	2:1^18^; 2:2^40^
PduT:PduS	6:1	6:1	
PduO:PduS	3:1	3:1	
PduP:PduQ	3:2	2:1	2:1^23^
Shell proteins:internal enzymes	4.6:1	6.2:1	
PduBB′ (WT:Δ*pduA*)	5:1		
PduJ (WT:Δ*pduA*)	2:3		
PduCDE (WT:Δ*pduA*)	2:1		

^a^The number of PduN was set to 1, and the ratio of other shell proteins was calculated accordingly.

^b^For comparison, the corresponding values for WT that were reported previously are listed.

Of the catalytic proteins for the WT Pdu MCP, PduCDE diol dehydratase was the most abundant. The subunit ratio of PduC, D, and E in the diol dehydratase was approximately 1:1:1 (Table 2), consistent with the stoichiometry revealed by the crystal structure of diol dehydratase in *Klebsiella oxytoca*^40^ but unsupportive to the ratio of 2:1:2 estimated previously^18^. The ratio of diol dehydratase reactivase PduG and PduH subunits was 2:1, consistent with an earlier *S*. Typhimurium study^18^, but distinct from the ratio of 2:2 in *K. oxytoca* estimated by SDS-PAGE densitometry^41^. The PduS enzyme, cobalamin reductase, was reported to interact with the PduO adenosyltransferase and shell protein PduT trimers^42,43^. Here we showed that the ratios of PduT:PduS and PduO:PduS were 6:1 and 3:1, respectively. PduP aldehyde dehydrogenase and PduQ alcohol dehydrogenase could potentially bind with each other^23^; our data revealed that PduP and PduQ made up 1.8% and 1.2% of total Pdu protein, respectively, at a ratio of 3:2 (Table 2), which is slightly lower than the ratio of 2:1 estimated in prior studies^23^. PduL had 33 copies per Pdu MCP, less than the content of the vertex protein PduN, confirming that this protein was a minor component of the Pdu MCP^24^.

### Variability of the stoichiometric organisation of Pdu MCPs

PduA has been reported to mediate the majority of interactions with other shell proteins, including PduB, PduJ, PduK, and PduU^29^. Comparison of protein stoichiometry of the WT and Δ*pduA* Pdu MCPs indicated that the absence of PduA resulted in an ~10% reduction in the total copy numbers of shell proteins (decline from 9607 to 8602, *p* = 0.045) and an ~41% reduction in the internal enzymes (decrease from 2065 to 1376 molecules, *p* = 0.005) (Fig. 3a, Table 1). The lower abundance of individual proteins was consistent with the reduced size of the Δ*pduA* Pdu MCPs as revealed by EM (Fig. 1c, Supplementary Fig. 3).

In the Δ*pduA* Pdu MCP, the ratio of individual shell proteins (B:B’:J:K:M:N:T:U) was 3:3:120:10:1:1:5:2, which was different from the ratio of 11:14:87:9:1:1:5:2 determined in the WT form (Table 2). There was a 38% increase in PduJ abundance within the Pdu MCP that lacked PduA (Fig. 3a, Table 2). Interestingly, the copy number of PduJ within the Δ*pduA* Pdu MCP (*n*_m_ = 1200 ± 148) was approximately equal to the total abundance of PduA (*n*_m_ = 307 ± 17) and PduJ (*n*_m_ = 869 ± 72) within the WT Pdu MCP (Fig. 3a, Table 1). We speculate that PduJ could compensate for the defects in the Pdu MCP structure caused by the lack of PduA, because PduA and PduJ proteins share high sequence (83%) and structural similarities^44^. In contrast, the PduBB’ content per Pdu MCP that lacked PduA decreased by a factor of 4, and the ratio of PduB and PduB’ in both complexes remained at 1:1 (Fig. 3a, Table 2), raising the possibility that PduA and PduBB’ were structurally coordinated in the shell^13,29^. The absence of PduA caused no significant difference in the abundance of PduK (*p* = 0.096), PduU (*p* = 0.146), PduM (*p* = 0.333), and PduT (*p* = 0.514).

In addition to the variation of shell protein abundance, the deletion of PduA also led to a 50% decline in the PduCDE content and an approximately 20% reduction in the content of PduO and PduQ (Fig. 3b). The PduP stoichiometry in the WT and Δ*pduA* Pdu MCPs was unchanged (Fig. 3b), which does not confirm the suggestion that PduP was encapsulated within the Pdu MCP via interactions with the C-terminus of PduA^45^. Significantly, the enzyme assays revealed no notable difference in the enzyme activities of the isolated WT and Δ*pduA* Pdu MCPs, when normalised by the total protein content (Fig. 1e). However, the reduced total protein content and PduCDE amount per Δ*pduA* Pdu MCP led to the estimation that the catalytic activity per Δ*pduA* Pdu MCP is lower than that per WT Pdu MCP, due to the reduction in cargo enzyme content as well as the altered shell organisation and permeability in the absence of PduA.

In summary, our results demonstrate that the stoichiometric composition and structure of the functional Pdu MCP can be remodelled.

### Association of PduV, PduW, and PduX with the Pdu MCP

Several less abundant building proteins were also characterised in the WT and Δ*pduA* Pdu MCPs, PduV (7 copies per Pdu MCP), PduW (11 copies per Pdu MCP), and PduX (4 copies per Pdu MCP) (Table 1). However, given that this abundance was at the similar level of the PduA mass spectrometric background signal detected in the Δ*pduA* Pdu MCP (10 copies per Pdu MCP, compared to 1841 copies in the WT, Table 1), it was uncertain that these low-abundance proteins represent genuine components of the Pdu MCP structure. Thus, to validate the association of PduV, PduW, and PduX with the Pdu MCP, we fused enhanced green fluorescent protein (eGFP) to the C-terminus of each of the three proteins individually, expressed each from the chromosome of *S*. Typhimurium LT2 (Supplementary Fig. 1) and characterised intracellular localisation. Fluorescence tagging and live-cell confocal fluorescence imaging have been exploited in studying the composition, assembly, and biogenesis of Pdu MCPs^29^ and carboxysomes^46–50^ in vivo. In addition, one shell protein PduT and one internal enzyme PduP were fused with eGFP, and a *S*. Typhimurium LT2 strain expressing native eGFP was constructed as the localisation controls. The GFP-fused strains grew slightly slower than the WT in the presence of 1,2-PD at limiting levels of B_12_ (Supplementary Fig. 2).

Confocal fluorescence imaging identified discrete intracellular patches of PduV that resembled the locations of PduT and PduP, representing the typical distribution pattern of Pdu MCPs in vivo (Fig. 3c). In contrast, PduW that catalyses the conversion of propionyl phosphate to propionate was dispersed throughout the cytoplasm, comparable to the distribution of native eGFP, indicating that PduW had a cytosolic location in *S*. Typhimurium LT2. PduX is responsible for the conversion of L-threonine to L-threonine phosphate and plays a role in the synthesis of Ado-B_12_, a substrate for the PduCDE enzymes during 1,2-PD degradation. PduX is not required when *Salmonella* grows aerobically on the MIM medium with succinate as the carbon source^28^. The GFP fluorescence of PduX at the native chromosome locus was not detectable. We thus overexpressed PduX in *S*. Typhimurium LT2 while inducing the synthesis of Pdu MCPs. PduX::eGFP exhibited even distribution in the cytosol, similar to free eGFP. Taken together, our results reveal that PduV functions as a component of the Pdu MCP, while PduW and PduX are likely not encased within the Pdu MCP structure, suggesting that metabolism of propionyl phosphate and L-threonine phosphate may occur predominately in the cytosol and is not present within the Pdu MCP lumen.

## Discussion

**Fig. 4:**
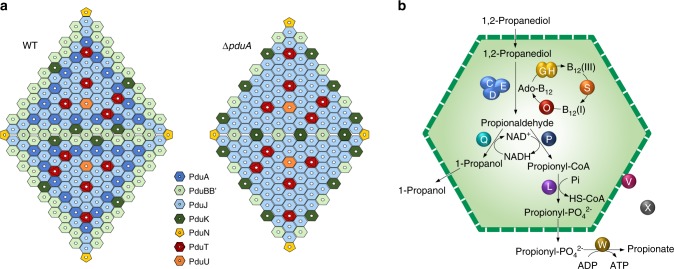
Schematic models of the Pdu MCP shell and interior organisations. **a** Models for two adjacent Pdu MCP shell facets in the *S*. Typhimurium LT2-WT and LT2-Δ*pduA*. Note that the model construction is based on the number of shell proteins from the absolute quantification data, and the relative geometric arrangement remains speculative. PduM is not shown here because of its unknown structure and uncertain role. **b** Model for the organisation of catalytic proteins. PduCDEGHLOPQV function as components of the Pdu MCP, whereas our confocal data suggest that PduW and PduX are not integrated into the Pdu MCP structure.

Despite endeavours to uncover BMC self-assembly and functions, their stoichiometry and overall structures are surprisingly unclear. This is mainly due to the difficulties to isolate intact, functional BMCs^37,51,52^ and the limitations of protein quantification. Numerous studies have used mass spectrometry to estimate the relative abundance of Pdu MCPs^18,37^ and carboxysomes^52,53^, which permitted comparison between different growth conditions but not the actual number of proteins per BMC. Recently, the use of single-molecule fluorescence microscopy allowed precise carboxysome protein stoichiometry to be determined by counting discrete bleaching steps of fluorescently tagged components within the carboxysome in live cyanobacterial cells^50^. Here we isolated Pdu MCPs using differential centrifugation to ensure their structural and functional integrity^36^, which was confirmed by EM, SDS-PAGE, and catalytical assays (Fig. 1). Absolute quantification using mass spectrometry, based on QconCAT technology^30,31^, was then used to characterise the isolated Pdu MCPs, leading to direct counting of the copy numbers of shell proteins and cargos per Pdu MCP, and to quantify the PduA-dependent stoichiometric changes in Pdu MCPs (Table 1). Our understanding of the levels of individual proteins led us to propose a model of the Pdu MCP structure (Fig. 4). The data show that QconCATs are a reproducible, broadly deployable tool that can be extended to study diverse BMCs and self-assembling biosystems.

We found that PduJ was the most abundant protein in both WT-MCPs (54.2%) and Δ*pduA*-MCPs (83.7%) isolated from *Salmonella*, consistent with previous observations from *Salmonella*^18^. In contrast, in the Pdu MCPs from a facultative anaerobic Gram-negative bacterium *Citrobacter freundii*, PduB’ was estimated to be the most abundant Pdu protein, accounting for 31% of all shell proteins, whereas the PduJ abundance was only 18%^37^. The variations probably indicate the species-dependent stoichiometric fingerprint of Pdu MCPs, although the genomic organisations of the *pdu* operons in the two organisms are highly conserved. The environment-mediated modulation of the protein content determined in carboxysomes^50^ also raises the possibility that the protein stoichiometry of Pdu MCPs may vary in different growth conditions, such as anaerobic environment and different carbon source, and this deserves further investigation.

Characterising the stoichiometric variations of BMCs allowed us to evaluate the inherent capacity of Pdu MCPs to remodel their composition and organisation for functional adaptation and to consider specific protein interactions in the polyhedral organelle. Our results demonstrated that the increased amount of PduJ proteins incorporated within the Δ*pduA* Pdu MCP corresponds to the amount of PduA in the WT Pdu MCP. We propose that the major shell proteins PduA and PduJ are functionally redundant and use a complementary strategy to retain the overall shape of Pdu MCPs.

The more thoroughly characterised carboxysomes could offer functional insights into the Pdu MCP, as multiple types of major shell homologues are also present in the α-carboxysome of *Halothiobacillus neapolitanus* (CsoS1A, B, and C)^54^, as does the β-carboxysome in *Synechocystis* sp. PCC 6803 (CcmK1 and CcmK2)^4^. In addition, the carboxysome minor shell homologues CcmK3 and CcmK4, which are encoded by the *ccmK3* and *ccmK4* genes that co-occur in cyanobacterial genomes, could form heterohexamers and are functionally linked^55^. The ratio of CcmK4:CcmK3 per carboxysome is relatively constant, in the range of 3.6–4.1, under different growth conditions^50^, validating their organisational correlation within the carboxysome shell. Whether the heterohexamer formation occurs in Pdu MCP shell proteins remains to be tackled.

In summary, we performed absolute protein quantification of the Pdu MCP metabolosome to characterise the stoichiometric and structural variations of Pdu MCPs with a combination of QconCAT-driven quantitative mass spectrometry and microscopic imaging. The growing interest in reprogramming BMCs for various biotechnological applications necessitates a deeper understanding of the protein stoichiometry and regulatory organisation of natural BMCs. Advanced knowledge about Pdu MCP self-assembly and the analytic methods developed here could be relevant to other self-assembling bacterial organelles and biological systems and so stimulate the engineering of bespoke, functional BMC structures in heterogeneous organisms for the enhancement of cellular metabolism.

## Methods

### Bacterial strains and growth conditions

The bacterial strains were derivatives of *S. enterica* serovar Typhimurium LT2^56^. The rich medium used was LB-Lennox medium (10 g L^−1^ tryptone, 5 g L^−1^ yeast extract, 5 g L^−1^ sodium chloride), and the minimal medium used was no-carbon-E (NCE) medium^57^. MIM is NCE medium supplemented with 1 mM MgSO_4_, 0.5% succinate, 50 μM Fe(III) citrate, and 0.6% 1,2-PD^36,58^. For Pdu MCP purification, overnight culture was sub-inoculated 1:100 in 20 mL of LB-Lennox medium and grown for about 2 h to an OD_600_ of 0.6–0.8. Subsequently, 4 mL of this LB culture was used to inoculate a 400 mL MIM culture, which was shaken aerobically at 37 °C until the OD_600_ reached 1.0–1.2.

### Construction of chromosomal mutations

*Salmonella* mutants (LT2-Δ*pduA*, LT2*-pduP*::*eGFP*, LT2*-pduT*::*eGFP*, LT2*-pduV*::*eGFP*, LT2*-pduW*::*eGFP*, and LT2*-pduX*::*eGFP*) were constructed by the gene disruption technique developed by Datsenko and Wanner^59^. A complete list of strains and plasmids used in this study and construction details could be found in Supplementary Table 1. Primers used in this study are listed in Supplementary Table 2. The template plasmids used for gene disruption and fluorescence fusion were pKD4 and pIJ786, respectively. pKD46 and pSIM5-*tet*^60^ were used for *lambda red* recombination. P22 transduction was used to move the individual mutations into a clean genetic background^61^. pCP20 plasmid, an ampicillin-resistant helper plasmid that expresses the FLP recombinase acting on the FRT sites flanking the resistance gene, was transformed into the bacteria by electroporation and used to eliminate the resistance cassette. All gene deletion mutants or fluorescently labelled strains were verified by PCR and DNA sequencing of PCR-amplified genomic DNA (Supplementary Fig. 1).

### Growth assays

Ten μL of an overnight LB cell culture was used to inoculate 10 mL of LB containing 0.6% 1,2-PD and shaken aerobically in a shaker at 220 rpm. After 6 h, cells were washed three times with the NCE medium supplemented with 0.6% 1,2-PD and 1 mM MgSO_4_. The washed and pelleted cells were resuspended in NCE medium (containing 0.6% 1,2-PD; 0.3 mM each of valine, isoleucine, leucine, and threonine; 50 μM ferric citrate; and 20 nM CN-B_12_) to an OD_600_ of 0.15, followed by growing at 37 °C in a microplate reader (BMG LABTECH) with intermittent shaking. The readings of OD_600_ were taken every hour. The growth curves were repeated at least three times in duplicate.

### Pdu MCP purification

The Pdu MCPs from both *S*. Typhimurium LT2-WT and LT2-Δ*pduA* were isolated by detergent treatment and differential centrifugation^36^. Briefly, 400 mL cells were harvested and washed twice with 40 mL of buffer A (50 mM Tris-HCl pH 8.0, 500 mM KCl, 12.5 mM MgCl_2_, and 1.5% 1,2-PD) and lysed with a mixture of 10 mL of buffer A and 15 mL of the bacteria-specific reagent (BPER-II, Thermo Fisher) supplemented with 5 mM 2-mercaptoethanol (Sigma-Aldrich), 1× protease inhibitor cocktail (PIC; Sigma-Aldrich), 25 mg lysozyme (Sigma-Aldrich), and 2 mg DNase I (Sigma-Aldrich), with 60 rpm shaking at room temperature for half an hour. Subsequently, Pdu MCPs were separated from cell debris by consecutive centrifuge steps at 4 °C (12,000 × *g* for 5 min twice to pellet cell debris, 20,000 × *g* for 20 min to pellet Pdu MCPs). The pellet after centrifugation at 20,000 × *g* was washed with a 10-mL mixture of buffer A and BPER-II (2:3, v/v) containing 1× PIC and then was resuspended in 0.2 mL of buffer B (50 mM Tris-HCl pH 8.0, 50 mM KCl, 5 mM MgCl_2_, 1% 1,2-PD) with 1× PIC. Finally, isolated Pdu MCPs were obtained by centrifugation at 12,000 × *g* (3 × 1 min) to further remove cell debris.

### PduCDE enzyme assay

PduCDE activity was measured by monitoring the formation of propionaldehyde^58^. Alcohol dehydrogenase was used as the coupled enzyme with the cofactor NADH. The components of the 200-μL reaction system included 0.5 μg of purified Pdu MCPs, 200 mM 1,2-PD, 25 μg mL^−1^ *Saccharomyces cerevisiae* alcohol dehydrogenase (Sigma-Aldrich), 0.4 mM NADH, 50 mM KCl, 50 mM HEPES buffer (pH 7.5), and 20 μM AdoB_12_. The reactions were plated in 96-well plates and were initiated by adding AdoB_12_. The absorbance (340 nm) was monitored at 37 °C with a microplate reader (BMG LABTECH). Different concentrations (0.1, 0.2, 0.3, and 0.4 mM) of NADH were used for generating standard curves to determine the amount of NADH consumption.

### SDS-PAGE analysis

Standard procedures for SDS-PAGE were employed. Protein composition was determined by 15% polyacrylamide gels and stained with Coomassie Brilliant Blue G-250.

### Transmission electron microscopy (TEM)

The Pdu MCPs structures in the LT2 WT and mutant strains were visualised using thin section^49^. *Salmonella* cells were pelleted and fixed with a mixture of 4% paraformaldehyde and 2.5% glutaraldehyde in 0.05 M sodium cacodylate buffer (pH 7.2) for 1 h. Samples were then post-fixed with 1% osmium tetroxide for 1.5 h, washed with ultrapure water, and stained with 1% uranyl acetate for overnight at 4 °C. Dehydration was conducted with a series of increasing alcohol concentrations (30–100%) before the cells were embedded in resin. Thin sections of 70 nm were cut with a diamond knife. The structures of isolated Pdu MCPs were characterised using negative staining TEM^52^. The isolated Pdu MCPs (1 mg mL^−1^) were stained with 3% uranyl acetate. Images were recorded using an FEI 120 kV Tecnai G2 Spirit BioTWIN TEM equipped with a Gatan Rio 16 camera. Image analysis was carried out using the ImageJ software. Statistical analysis was performed using Student’s *t* test conducted by IBM SPSS Statistics 24.

### Proteomic analysis

Absolute quantification for the Pdu MCP proteomics was conducted using concatenated signature peptides encoded by a QconCAT^31^. In general, two peptides, serving as surrogates, were nominated to quantify each protein based on the published design principles (Supplementary Table 4)^62^. Only one peptide meeting the criteria was selected for quantification of PduB, PduN, and PduX. The data are still reliable, given the excellent agreement between the nominated two peptides of other Pdu MCP proteins. All peptides were searched against the Uniprot of *S. enterica* database to make sure that they are unique. The QconCAT gene was subcloned into a plasmid vector (pEU01) optimised for CFS, provided by GeneMill, University of Liverpool. The coding DNA sequence and translated peptide sequence for QconCATs are shown in Supplementary Table 3. The CFS (WEPRO8240/8240H/8240G Expression Kit, CellFree Sciences) was used to express the QconCAT proteins labelled with stable isotopes^30^. Statistical analysis was performed using Student’s *t* test.

WT and PduA knockout Pdu MCPs preparations, 4 replicates each, were diluted to a final protein concentration of 2 μg in 25 mM NH_4_HC0_3_ (40 μL). QconCAT (approximately 10 pmol) was added, and samples were reduced by the addition of 2.5 μL of 12 mM dithiothreitol (Melford Laboratories) in 25 mM NH_4_HC0_3_ and incubation at 60 °C for 10 min. Alkylation was carried out by the addition of 2.5 μL of 36 mM iodoacetamide (Sigma-Aldrich) in 25 mM NH_4_HC0_3_ and incubation at room temperature for 30 min in the dark. Trypsin 2.5 μL (200 ng in 25 mM NH_4_HC0_3_; E:P 1:10) was added to each sample in a final digest volume of 50 μL. Trypsin Gold, mass spectrometry grade, was from Promega. Samples were incubated at 37 °C overnight. To remove residual Rapigest that was present in the QconCAT stock solution, digests were acidified by the addition of 0.5 μL of trifluoroacetic acid (TFA, Fisher Chemicals) followed by incubation at 37 °C for 45 min. Samples were centrifuged at 17,200 × *g* for 30 min and transferred to fresh low-bind tubes.

LC-MS analyses were conducted on a QExactive HF quadrupole-Orbitrap mass spectrometer coupled to a Dionex Ultimate 3000 RSLC nano-liquid chromatograph (Thermo Fisher, UK, Xcalibur software version 4.1). Sample digest (2 μL) was loaded onto a trapping column (Acclaim PepMap 100 C18, 75 µm × 2 cm, 3 µm packing material, 100 Å) using a loading buffer of 0.1% (v/v) TFA, and 2% (v/v) acetonitrile in water for 7 min at a flow rate of 12 µL min^−1^. The trapping column was then set in-line with an analytical column (EASY-Spray PepMap RSLC C18, 75 µm × 50 cm, 2 µm packing material, 100 Å), and the peptides were eluted using a linear gradient of 96.2% A (0.1% [v/v] formic acid):3.8% B (0.1% [v/v[formic acid in water:acetonitrile [80:20] [v/v]) to 50% A:50% B over 30 min at a flow rate of 300 nL min^−1^, followed by washing at 1% A:99% B for 5 min and re-equilibration of the column to starting conditions. The column was maintained at 40 °C, and the effluent was introduced directly into the integrated nano-electrospray ionisation source operating in positive ion mode. The mass spectrometer was operated in MS-only mode with survey scans between *m*/*z* 350–2000 acquired at a mass resolution of 240,000 (full width at half maximum (FWHM)) at *m*/*z* 200. The maximum injection time was 50 ms, and the automatic gain control was set to 3e^6^.

Glufibrinopeptide B (Glufib, Sigma-Aldrich) unlabelled standard peptide was diluted to 200 fmol L^−1^ in 0.1%(v/v) TFA/3% (v/v) acetonitrile and mixed with an equal volume of digest. Two μL was loaded on column and run on a short 10 min gradient of 96.2% A (0.1% [v/v] formic acid):3.8% B (0.1% [v/v[formic acid in water:acetonitrile [80:20] [v/v]) to 50% A:50% B over 30 min at a flow rate of 300 nL min^−1^, followed by washing at 1% A:99% B for 5 min and re-equilibration of the column to starting conditions. An MS-only method was run at a mass resolution of 70,000 (FWHM) at *m*/*z* 200 on a QExactive quadrupole-Orbitrap mass spectrometer coupled to a Dionex Ultimate 3000 RSLC nano-liquid chromatograph with a maximum injection time of 50 ms and the automatic gain control of 3e^6^.

To determine the absolute amount of QconCAT and surrogate analyte peptide in each digest, a known amount of unlabelled Glufib, was mixed in a 1:1 ratio with each digest and from the ratio of peak areas to a corresponding labelled quantification peptide was calculated using Skyline open source software the amount of Pdu peptides (femtomole) was determined^38^. An in-house Mascot Server search engine (version 2.6.2, Matrix Science) was used to search protein databases.

### Confocal microscopy

Overnight culture was sub-inoculated 1:100 in 5 mL of LB-Lennox medium for about 2 h to an OD_600_ of 0.6–0.8. Fifty μL of this LB culture was inoculated to 5 mL MIM shaking aerobically at 37 °C until OD_600_ reached 1.0–1.2. Then fluorescence strains were examined by confocal microscopy using a Zeiss LSM780 with an 63 × oil-immersion objective and excitation at 488 nm.

### Statistics and reproducibility

All experiments reported here were performed at least three times independently.

### Reporting summary

Further information on research design is available in the Nature Research Reporting Summary linked to this article.

## Supplementary information

Supplementary Information

Reporting Summary

## Data Availability

The source data underlying Table 1, Figs. 1e and 3a–b, and Supplementary Figs. 2, 3a, b, and 10 are provided as a Source Data file. The mass spectrometry proteomics data associated with Supplementary Figs. 6 and 11 have been deposited to the ProteomeXchange Consortium via the PRIDE partner repository with the data set identifier PXD015111. All data are available from the corresponding author upon request.

## Source Data

Source Data
