# Supplementary material for: Decoding the stoichiometric composition and organisation of bacterial metabolosomes
Source: Nat Commun. 2020 Apr 24;11:1976. doi: 10.1038/s41467-020-15888-4 (PMC7181861; doi:10.1038/s41467-020-15888-4)
Supplement: Supplementary file 1 — Supplementary Information [file 41467_2020_15888_MOESM1_ESM.pdf]

Supplementary Information

for

Yang et al., "Decoding the stoichiometric composition and organisation of bacterial metabolosomes"

Supplementary Table 1. Strains of *S.* Typhimurium LT2 derivatives and plasmids. Relevant antibiotic resistances are indicated by ^{R}: Ap, ampicillin; Km, kanamycin, Am, apramycin; Cm, chloramphenicol.

strains/plasmids	Description	Reference/origin
**LT2 derivatives:**		
LT2	LT2, WT	[1]
LT2-Δ*pduA*::*kan*	Δ*pduA*; Km^R	This study
LT2-Δ*pduA*	Δ*pduA*	This study
LT2-*pduP*::*eGFP*::*apr*	LT2 derivative with PduP fused with eGFP; Am^R	This study
LT2-*pduP*::*eGFP*	LT2 derivative with PduP fused with eGFP	This study
LT2-*pduT*::*eGFP*::*apr*	LT2 derivative with PduT fused with eGFP; Am^R	This study
LT2-*pduT*::*eGFP*	LT2 derivative with PduT fused with eGFP	This study
LT2-*pduV*::*eGFP*::*apr*	LT2 derivative with PduV fused with eGFP; Am^R	This study
LT2-*pduV*::*eGFP*	LT2 derivative with PduV fused with eGFP	This study
LT2-*pduW*::*eGFP*::*apr*	LT2 derivative with PduW fused with eGFP; Am^R	This study
LT2-*pduW*::*eGFP*	LT2 derivative with PduW fused with eGFP	This study
LT2-*pduX*::*eGFP*::*apr*	LT2 derivative with PduX fused with eGFP; Am^R	This study
LT2-*pduX*::*eGFP*	LT2 derivative with PduX fused with eGFP	This study
**Plasmids:**		
pKD4	*aph*-cassette template plasmid;Km^R	[2]
pKD46	*λ* Red recombination plasmid, arabinose-inducible; Ap^R	[2]
pCP20	Plasmid carrying the Flp recombinase to remove kanamycin resistance from pKD13 derived resistance cassette insertions; Ap^R	[2]
pSIM5-*tet*	λ Red recombination plasmid, temperature-inducible; Tc^R	[3]
pIJ786	*eGFP*-cassette template plasmid; Am^R	PBL Biomedical Laboratories
pXG10-eGFP	Plasmid for expression of eGFP; Cm^R	This study
pXG10-PduX::eGFP	Plasmid for expression of PduX::eGFP; Cm^R	This study
pEUE01-QconCATs	Plasmid carrying the synthesized DNA sequence for QconCATs; Ap^R	This study

Supplementary Table 2. Primers used in this study. The primers (bold) contain both priming sites and homology extensions for amplifying relative genes and recombination, and the others are segregation primers which are about 150 bp upstream and downstream the target genes for PCR verification and sequencing.

Primers	Sequence (5'→3')
pduA_del_F	**TCTTATAGTCCCAACTATCGGAACACTCCATGCGAGGTCTTTATGGTGTAGGCTGGAGCTGCTTC**
pduA_del_R	**GTTCCACCAGCTCATTGCTGCTCATTGGCTAATTCCCTTCGGTAACATATGAATATCCTCCTTAG**
pduA_up	AAATATTGCACAAGCCAACTTATC
pduA_down	GGCCCAGGGTATCGCCAATG
PduP_GFP_F	**TCCCGGCGCTGCGTACTGACCAACGGCTTTTCTATTCGCCTGCCGGGCCCGGAGCTGCC**
PduP_GFP_R	**TTTGTAGTGAGAAGGTATTCATCGCGACCTCAGTTAGCGATTCCGGGGATCCGTCGACC**
PduP_GFP_up	TGCAAACCTCGATATTCGTC
PduP_GFP_down	GTTATCTCGCTAAAGACGCT
PduT_GFP_F	**CGCCCGCATGAAGCCATGTGGCGACAGATGGTGGAGGGGCTGCCGGGCCCGGAGCTGCC**
PduT_GFP_R	**TTGTCGGTTGTCTTTCCATTACCCCTCCACCATCTGTCGATTCCGGGGATCCGTCGACC**
PduT_GFP_up	TGCTACATGGTGGTGGCGGG
PduT_GFP_down	TGCTTCGCTCGGCGTGATGG
PduV_GFP_F	**GATTTTCTGAATTCGAAGGAACCTTTATGTCTTACAAAACTGCCGGGCCCGGAGCTGCC**
PduV_GFP_R	**TAATGGCCATTATTTTGTAAGACATAAAGGTTCCTTCGAATTCCGGGGATCCGTCGACC**
PduV_GFP_up	TATTTCGCTGGTTGCGGAGT
PduV_GFP_down	GCGTCTTAATTGTCACCTGC
PduW_GFP_F	**ATTGCGCTACCGGCCACGGAAGGGCTTTGTGTACCAGCCCTGCCGGGCCCGGAGCTGCC**
PduW_GFP_R	**TTATTACCATGACTTGCCGCAATGCGTCGTATAACCCTCATTCCGGGGATCCGTCGACC**
PduW_GFP_up	TGCGACATTTATCCAGACGG
PduW_GFP_down	TGGATAAGTTCCCCGCATGA
PduX_GFP_F	**CATTTACTCAAGATGGTCACTGGCGGGGTCAAACTGCAGCTGCCGGGCCCGGAGCTGCC**
PduX_GFP_R	**GAATAATGCGCTACGGCAGTGGGAGCGTATATCAGGCTCATTCCGGGGATCCGTCGACC**
PduX_GFP_up	TAGCGGTAGCGTGGTGGGTC
PduX_GFP_down	CTCCAAAGAGCGTCGCGACA

Supplementary Table 3. DNA and protein sequences of the synthetic Pdu QconCAT peptide

DNA sequence (2067 bp)

atgggcacccgcatgggaactcgcgagggcgttaacgacgaggaaggattcttcagcgccaggtacgagaa
gatcggctctggcctcgtgaccgtgatcgttaggggcgacgtgatggtgaagtccgccaacgttatgctcg
tgggctatgagaagattggcagcattgcccgcgtggcgaccccagagcaacaagctatcccaggacaacca
cagccaatccgcgagactgccagataccgctctatcggaatcctcggagctaggactggcgctactgccag
ggcttcttacgccctcgagaaggctttcggaatcgcccgctacggcatcaacctcaacagggccgaagagg
acatcaagttcgcccaagagatcatcaacaagaaccgcaacatcctccgcgaggtgatcgccggaattgag
gaagagggaatcaaggccagggtgtgcttcaagtcctccgacgttgccttcgtggccgtggaaggcaatag
gctctctggcgacatgaggatcaccccagagactctcaggctccaagcctctgccaaggtgagcgattacc
cactcgccaacaagcacccagagctgcaaagagatgacggcgtcctcgtgtccaacaggctcgagaagaag
gaaagggttttcgtcaccaacgcgctgagggcccttagggccgccagatcttctccactcctcgttggaat
cgcctgcgacaggcattctctctgcgataggcatagcctcgtggtgcactacaagaacctgccaatggtca
agagcgccaacgtgcaactcgtcggctacgaaaagatcgggtcctacgagaagattggaagcggcctcgtc
accgtcatggtgcgcggagatgttatggccaagtccgcctctatcaccctgctcgccctcgaaaagaccaa
cggcctggaaaagactaatggctccggctggatggtgatcaagatcaccggaccactccgctccgtgagcc
aagtcgagatctctaggaccgacgccgatgccaggactctcggaattgctgccccactcaggatgtccggc
ctccaaaggatcgtggaagagatcgtgtccaggctccacaggtcctacagggatgttgccttgctctctcg
cggaagggttctcgccagagttactggcgccgtggtgagcacccaaaagtccccatctatcaaccgcctct
ccgactgcctctacgctctcgctagagccgaagatatggccagggtcgagccactccacagcttcattctt
ccaggacgctgcgaggccgccgagaagtctcttatcgtggttgagtctgtggccgagaggctcgttcaagc
cgctaggaccctccaaacctccatcttcgtgaagaacggcccatctccaagggcctccgacttcactgatg
ccctcgctgagaaggccgcgaagaagcaacgctgcgttctcccatctctcgccgtggctctcaaggaaggt
agggacgagaggaccgtgctctctgttgctaggaccgtgtgccaatgcaggctctgcactgatctctgccc
aaggcatctgatcgccgtgaagggctctaacgtgaccctcgtgagggttcacatgggcgagaagggactgc
tcgtgtacagatccgtgatcgccactaagtcaggcgccgtcgagatcggcttcctcgataggttcaccggc
ctcgatcgcttcactggcgctgttgttctcaccggcgacgtttccgctgttgagtacgccctcaagcaagt
gacctgcggcaagacctctctcacccaatctctcagaggcgaggccgtgactaaggccgatcttgctgagc
cacaaaggatctccctcgaccacagggacgccgttactctcctgcttgagaagctcctcggctcccacaag
tacgtgtcaggcgttctggccgagaagctgggcgttgacgttaggtctagcatcccagtggccaagggcat
ggccactctcaggacccaagactaccataggctcccaaggctcgccgctgctctcgagcaccatcaccatc
atcattag

Protein sequence (688 AA)

MGTRMGTREGVNDEEGFFSARYEKIGSGLVTVIVRGDVMVKSANVMLVGYEKIGSIARVATPEQQAIPGQP
QPIRETARYRSIGILGARTGATARASYALEKAFGIARYGINLNRAEEDIKFAQEIINKNRNILREVIAGIE
EEGIKARVCFKSSDVAFVAVEGNRLSGDMRITPETLRLQASAKVSDYPLANKHPELQRDDGVLVSNRLEKK
ERVFVTNALRALRAARSSPLLVGIACDRHSLCDRHSLVVHYKNLPMVKSANVQLVGYEKIGSYEKIGSGLV
TVMVRGDVMAKSASITLLALEKTNGLEKTNGSGWMVIKITGPLRSVSQVEISRTDADARTLGIAAPLRMSG
LQRIVEEIVSRLHRSYRDVALLSRGRVLARVTGAVVSTQKSPSINRLSDCLYALARAEDMARVEPLHSFIL
PGRCEAAEKSLIVVESVAERLVQAARTLQTSIFVKNGPSPRASDFTDALAEKAAKKQRCVLPSLAVALKEG
RDERTVLSVARTVCQCRLCTDLCPRHLIAVKGSNVTLVRVHMGEKGLLVYRSVIATKSGAVEIGFLDRFTG
LDRFTGAVVLTGDVSAVEYALKQVTCGKTSLTQSLRGEAVTKADLAEPQRISLDHRDAVTLLLEKLLGSHK
YVSGVLAEKLGVDVRSSIPVAKGMATLRTQDYHRLPRLAAALEHHHHHH*

Supplementary Table 4. Peptide sequences determined by MS for Pdu protein quantification. Two peptides, serving as surrogates, were nominated to quantify each protein based on the design principles previously described [4]. One peptide was selected for PduB, PduN, and PduX, because only one peptide meets the QconCAT design criteria. The peptide with higher value was used for quantification (shown in bold), reasoning that signal loss from endogenous peptide is more likely. * The amount of PduB was calculated using the differential abundance of PduB and PduB/B'.

Protein	Nominated peptides
PduA	IGSGLVTVIVR
	**SANVMLVGYEK**
PduB *	**VATPEQQAIPGQPQPIR**
PduB/B'	SIGILGAR
	**ASYALEK**
PduC	**YGINLNR**
	FAQEIINK
PduD	EVIAGIEEEGIK
	**SSDVAFVAVEGNR**
PduE	**ITPETLR**
	VSDYPLANK
PduG	**DDGVLVSNR**
	VFVTNALR
PduH	SSPLLVGIACDR
	**HSLVVHYK**
PduJ	**SANVQLVGYEK**
	IGSGLVTVMVR
PduK	SASITLLALEK
	**TNGSGWMVIK**
PduL	**SVSQVEISR**
	TLGIAAPLR
PduM	**IVEEIVSR**
	DVALLSR
PduN	**VTGAVVSTQK**
PduO	LSDCLYALAR
	**VEPLHSFILPGR**
PduP	**SLIVVESVAER**
	TLQTSIFVK
PduQ	**ASDFTDALAEK**
	CVLPSLAVALK
PduS	**TVLSVAR**
	LCTDLCPR
PduT	GSNVTLVR
	**GLLVYR**
PduU	**SGAVEIGFLDR**
	FTGAVVLTGDVSAVEYALK
PduV	**TSLTQSLR**
	ADLAEPQR
PduW	**DAVTLLLEK**
	YVSGVLAEK
PduX	**SSIPVAK**

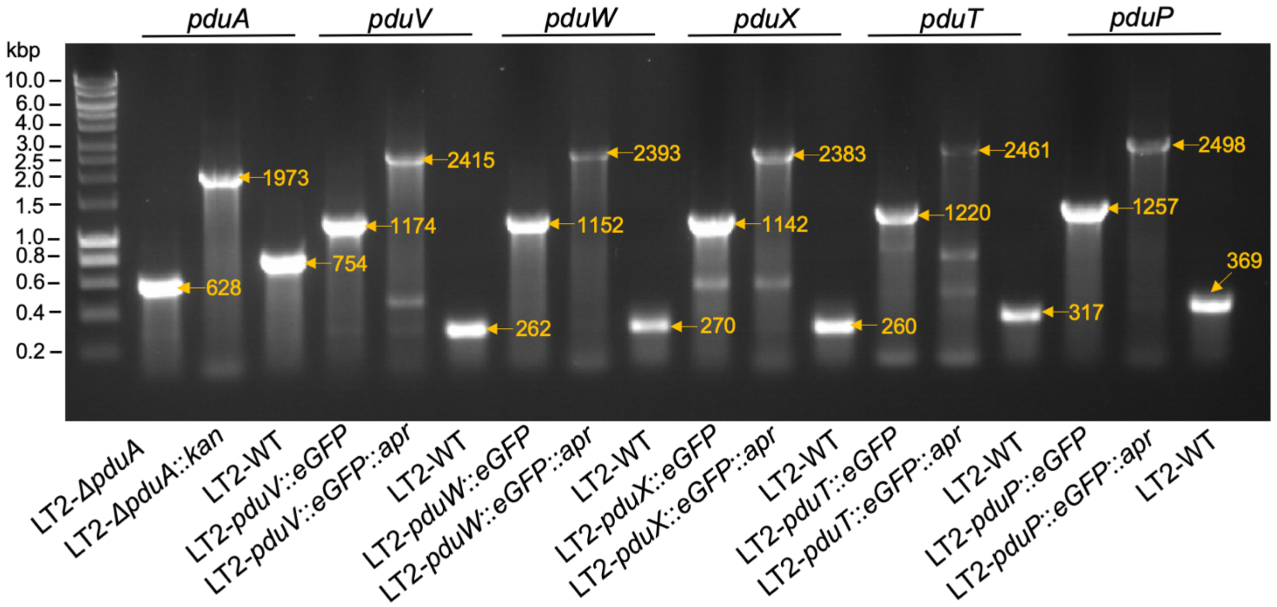

Supplementary Fig. 1. PCR-based confirmation of LT2-Δ*pduA*, LT2-*pduP*::*eGFP*, LT2-*pduT*::*eGFP*, LT2-*pduV*::*eGFP*, LT2-*pduW*::*eGFP*, *and* LT2-*pduX*::*eGFP*. The sizes (bp) of the PCR products were indicated (yellow).

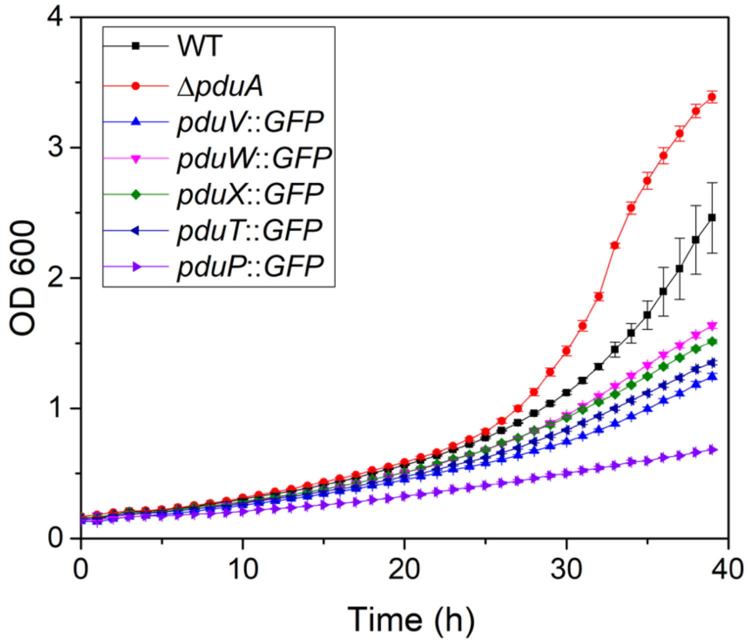

Supplementary Fig. 2. Growth assay for the WT and Δ*pduA* and fluorescence-labelling strains under biotin-limiting conditions. Cells were growing aerobically at 37°C in the NCE medium, supplemented with 0.6% 1,2-PD; 0.3 mM each of valine, isoleucine, leucine, and threonine; 50 μM ferric citrate; 20 nM CN-B_{12}. The values were presented as the means and SD (error bar, n = 3). n, number of biologically independent samples. Source data are provided as a Source Data file.

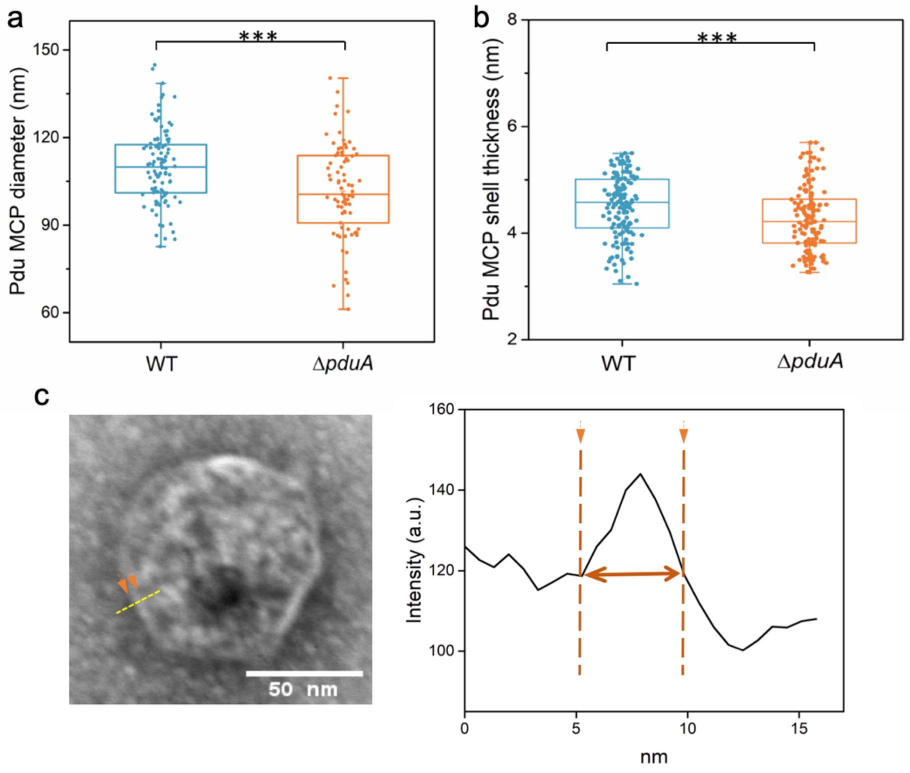

Supplementary Fig. 3. Diameter and shell thickness of Pdu MCP in *S.* Typhimurium LT2 measured from TEM images. (a) and (b) The WT-Pdu MCP has a mean diameter of 110 ± 13 nm (n = 104) and the shell thickness of 4.5 ± 0.6 nm (n = 156). The Δ*pduA*-Pdu MCP has a mean diameter of 102 ± 16 nm (n = 87) and the shell thickness of 4.3 ± 0.6 nm (n = 141). The diameter and shell thickness of the WT-Pdu MCP are significantly different from those of the Δ*pduA*-Pdu MCP (p = 1.12e^{-4} and 0.001, respectively, two-sided t-test). Each measurement of the diameter is the mean of the three vertex-to-vertex measurements from a single Pdu MCP (n represents the number of particles measured) as described previously for carboxysomes [5]. Boxplot centre lines correspond to the median value; upper and lower hinges correspond to the 75th and 25th percentiles; and upper and lower whiskers extend from the box to the largest or samllest value correspondingly, but no more than 1.5 times the interquantile range. Error bars represent SD. (c) Measurement of the Pdu MCP shell thickness on EM images. Cross-section profile reveals the shell thickness of 4.5 nm. n, the number of edges measured. Source data are provided as a Source Data file.

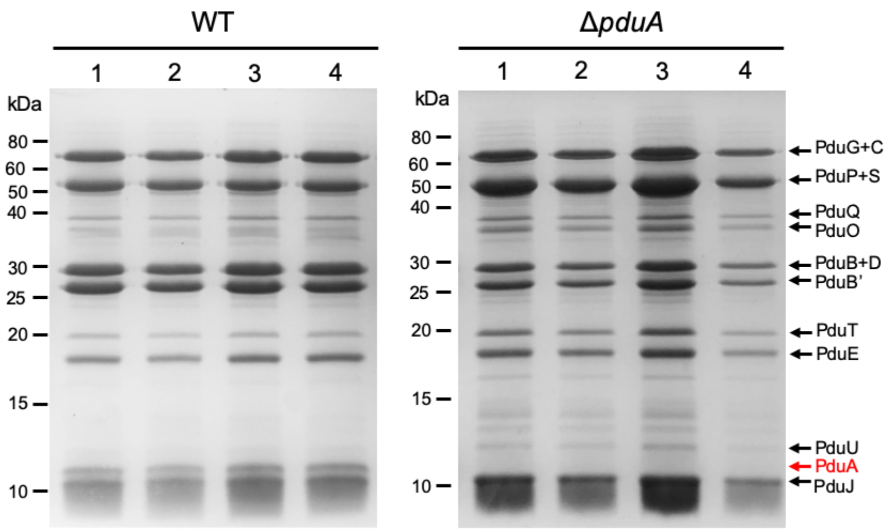

Supplementary Fig. 4. SDS-PAGE of purified Pdu MCPs from the WT and Δ*pduA* cells with four biological replicates prepared for quantification by QconCAT. The PduA bands (red) are absent in the Δ*pduA* samples.

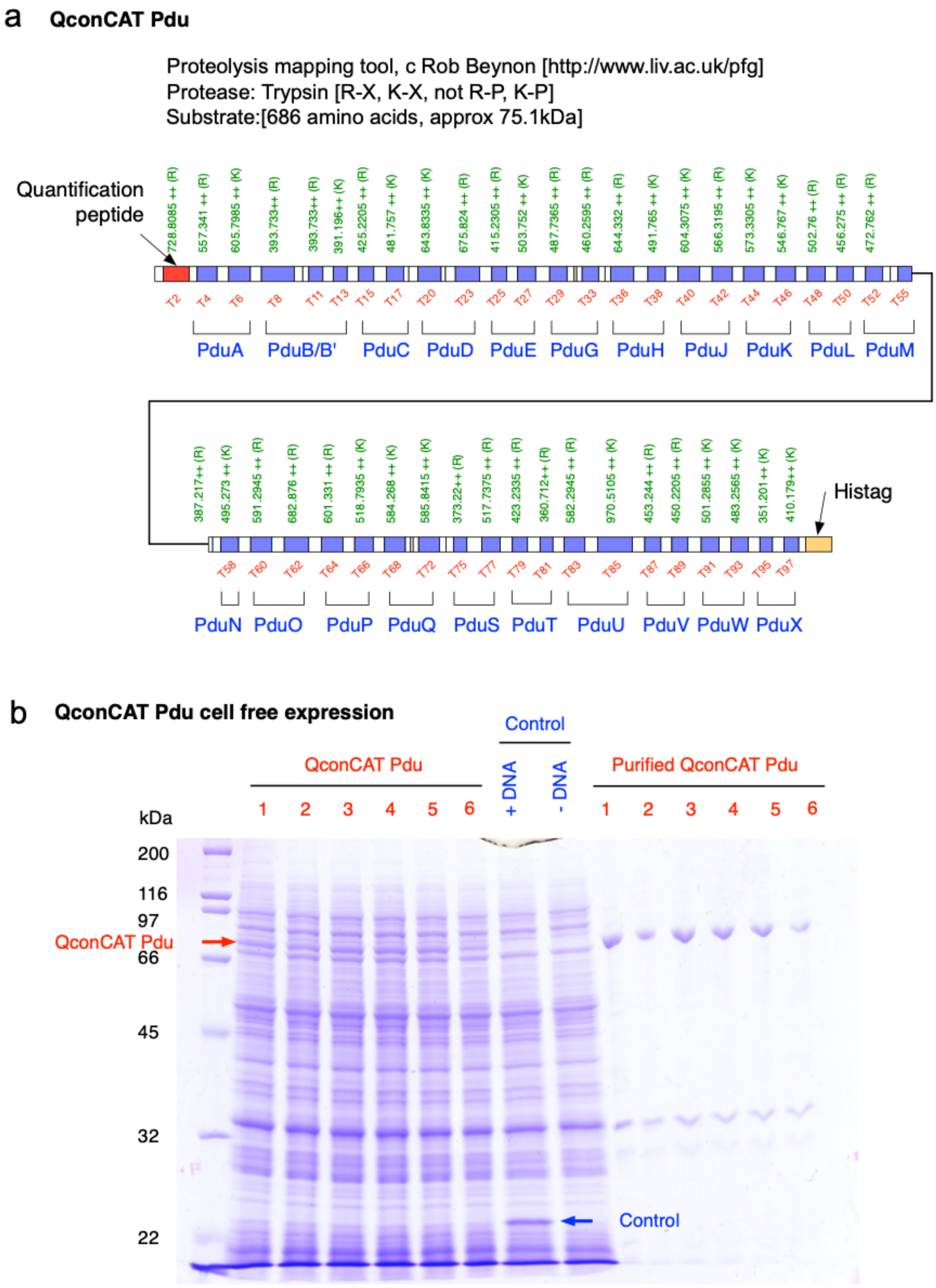

Supplementary Fig. 5. Structure and expression of the Pdu QconCAT. (a) A schematic representation of the quantification concatamer, for quantification of 22 Pdu proteins. 41 quantification peptides in QconCAT Pdu are represented by blue boxes. Mass values for the doubly-charged peptide ion for the unlabelled QconCAT are aligned above each peptide (green text). The AQUA (absolute quantification) peptide, and the hexahistidine tag for QconCAT purification are shaded in red and yellow respectively. (b) SDS-PAGE analysis of QconCAT expression and purification. The gene encoding QconCAT Pdu was sub-cloned into the cell-free expression vector pEU-E01-MCS. QconCAT Pdu was prepared by wheat germ cell-free synthesis in the presence of [$^{13}C_6$,$^{15}N_4$]arginine and [$^{13}C_6$,$^{15}N_2$]lysine and purified by a hexahistidine tag. Replicate reactions were carried out and the purified QconCAT pooled. The negative control (- DNA) contained empty pEU01 plasmid and the positive control (+ DNA) contained DHFR encoded in the pUE01 plasmid vector.

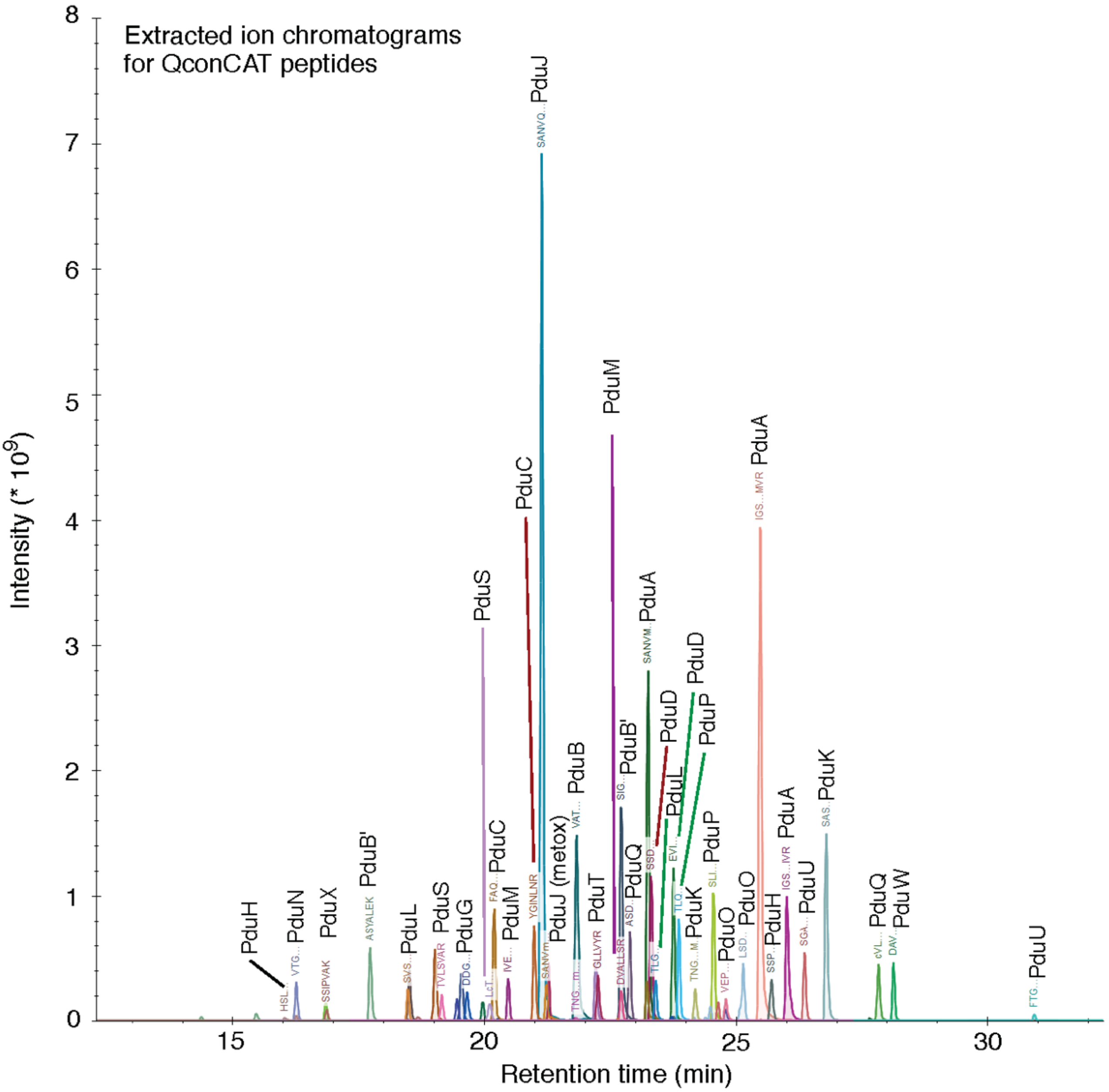

Supplementary Fig. 6. LC-MS profile of Pdu QconCAT peptides. Stable isotope-labelled QconCAT Pdu was digested in-solution with trypsin and the digest analysed on a 1h LC-MSMS method. The raw data file was uploaded into Skyline and the QconCAT peptide masses extracted.

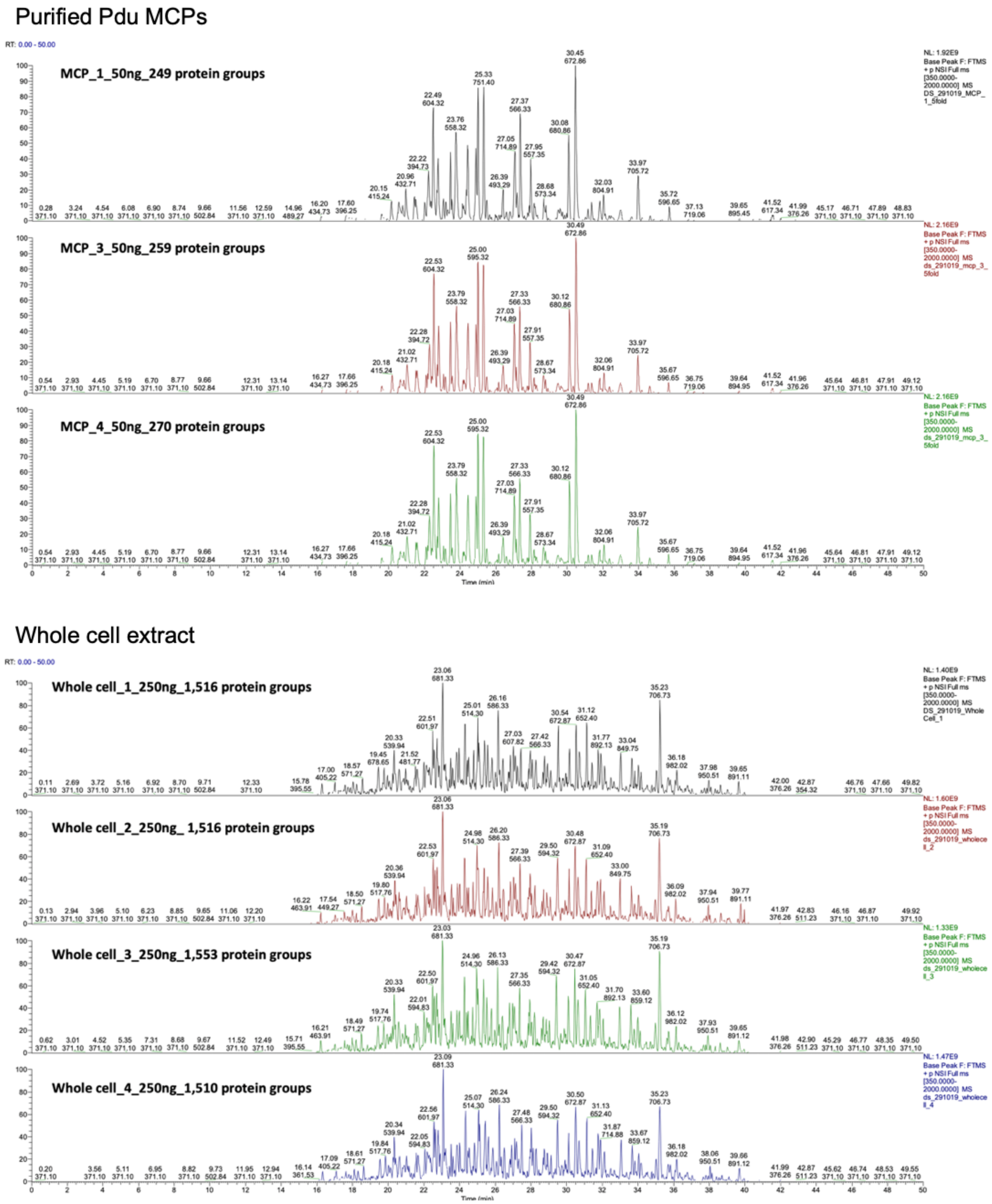

Supplementary Fig. 7. Base peak chromatograms for purified Pdu MCPs and whole-cell extracts. Three purified Pdu MCPs and four whole-cell extracts were analysed in DDA mode. The base peak intensities were very similar, as expected from the SDS-PAGE data of the samples. Further, the total numbers of identified protein groups were very similar within each biological replicate, and as anticipated, the number of protein groups was considerably higher in the cell extract than in the Pdu complex samples. The simplicity of the base peak chromatogram of the complexes is further confirmation of the use of high resolution, MS1-based quantification using QconCATs.

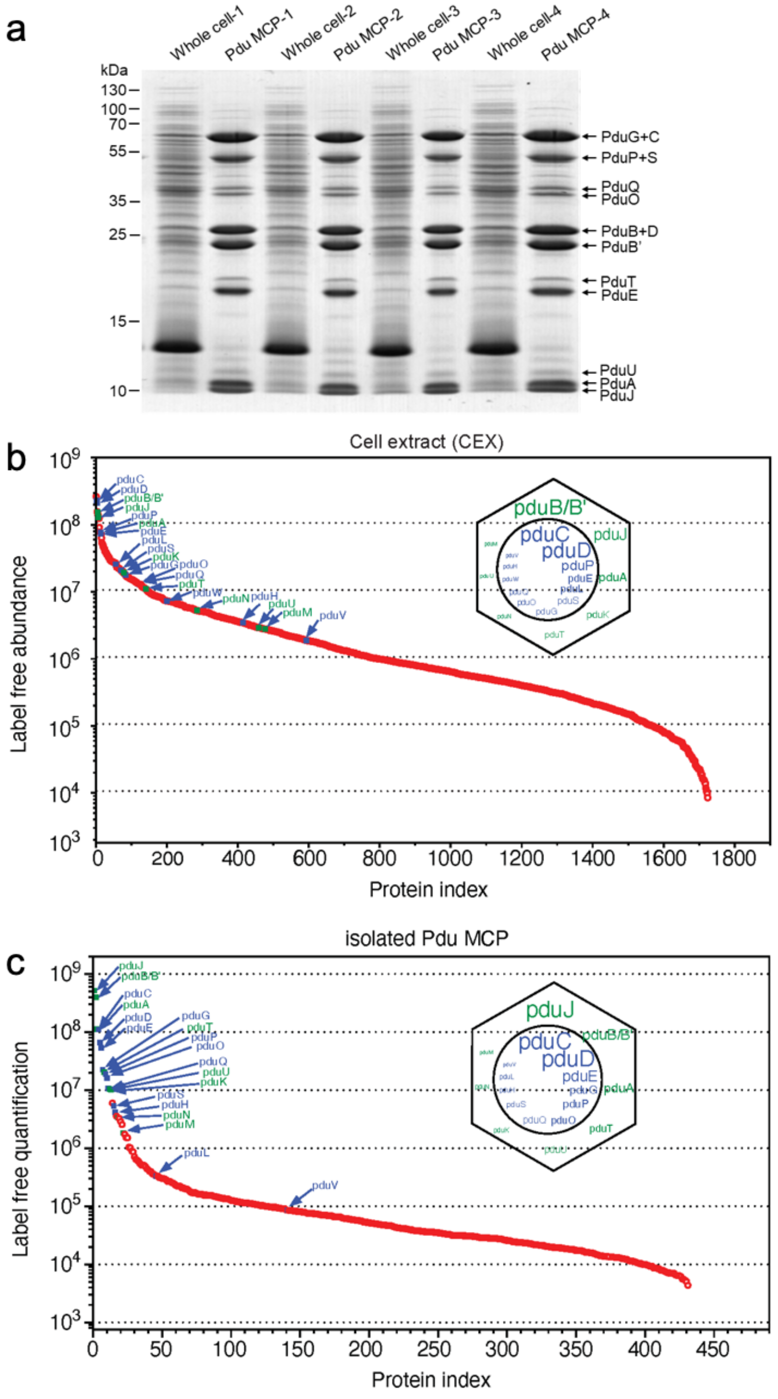

Supplementary Fig. 8. Label-free quantification of MCP in cell extracts and purified complexes. Samples of WT broken cell extracts (CEX) or of purified Pdu MCP complexes were analysed by LC_MS/MS and label-free quantification. Four samples were analysed. (a) SDS-PAGE of the cell extracts and purified complexes were resolved by SDS-PAGE to illustrate the consistency of the preparations and the extreme enrichment of the purified Pdu MCP. (b and c) Proteins from cell extracts and isolated Pdu MCPs were discriminated according to whether they are shell (green) or enzyme (blue) components. Each Pdu protein was quantified using label-free approaches, and the proteins are ranked according to their abundance. As expected, there were many more proteins in the cell extracts. The Pdu proteins are then highlighted. The results show explicitly the considerable enrichment of Pdu MCP proteins in the isolated sample.

PduB’ (ASYALEK)

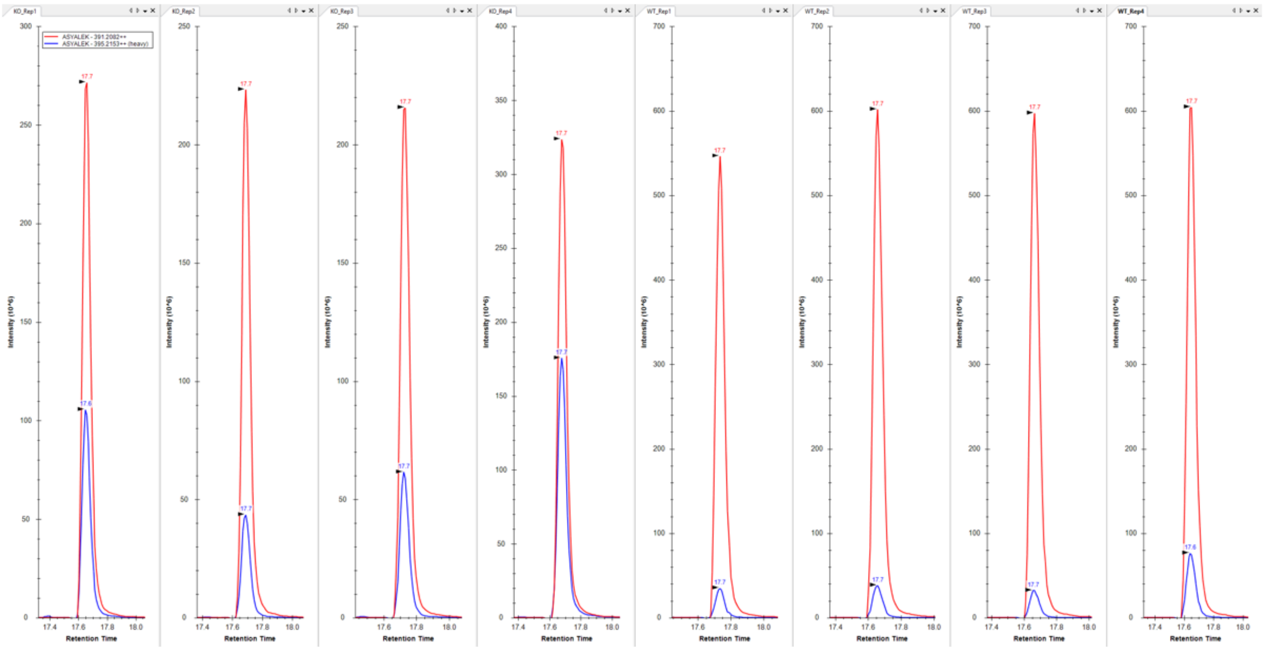

PduA (IGSGLVTIVR)

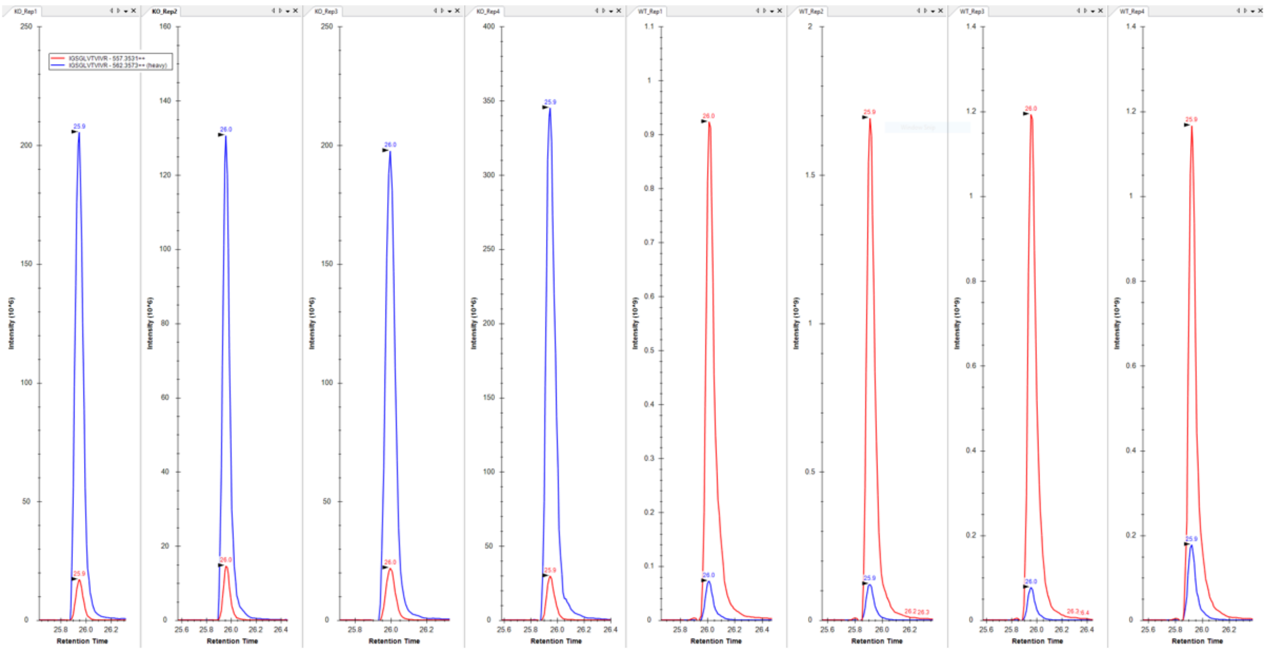

Supplementary Fig. 9. Extracted ion chromatograms for PduA peptides. For two peptides; top panel: peptide is from PduB’ (ASYALEK), bottom panel: PduA (IGSGLVTVIVR) which is knocked out in the mutant. The ‘light’ purified complexes are defined by the red traces and the ‘heavy’ (QconCAT standard) is the blue trace. Marked reduction of the deleted protein PduA is evident from the inverted traces for IGSGLVTVIVR.

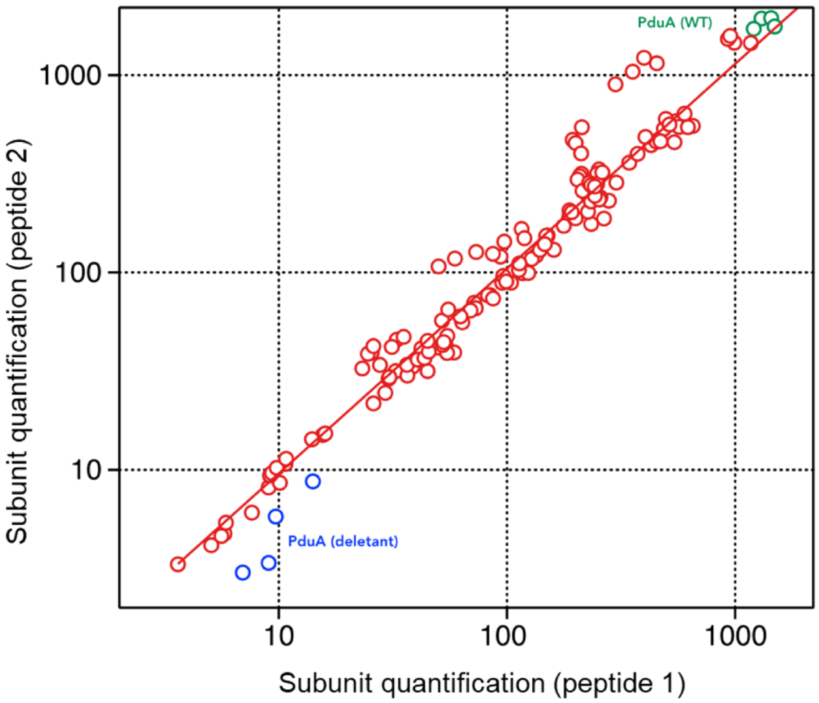

Supplementary Fig. 10. Agreement between two QconCAT peptides used in quantification. For each protein, for each replicate and WT and PduA deletant, the absolute quantification values derived from one quantotypic peptide are plotted against the second quantotypic peptide for the same protein (the peptides are randomly assigned according to whether the quantification values were higher in one than the other). These data illustrate the excellent concordance between the values obtained from both peptides. The values for PduA obtained with the WT (green) and PduA deletant (blue) are highlighted to emphasise the degree of reduction in this protein. The values of the peptides were normalised by the estimated copy number of PduN peptides per Pdu MCP particle, 60 (Table 1). Source data are provided as a Source Data file.

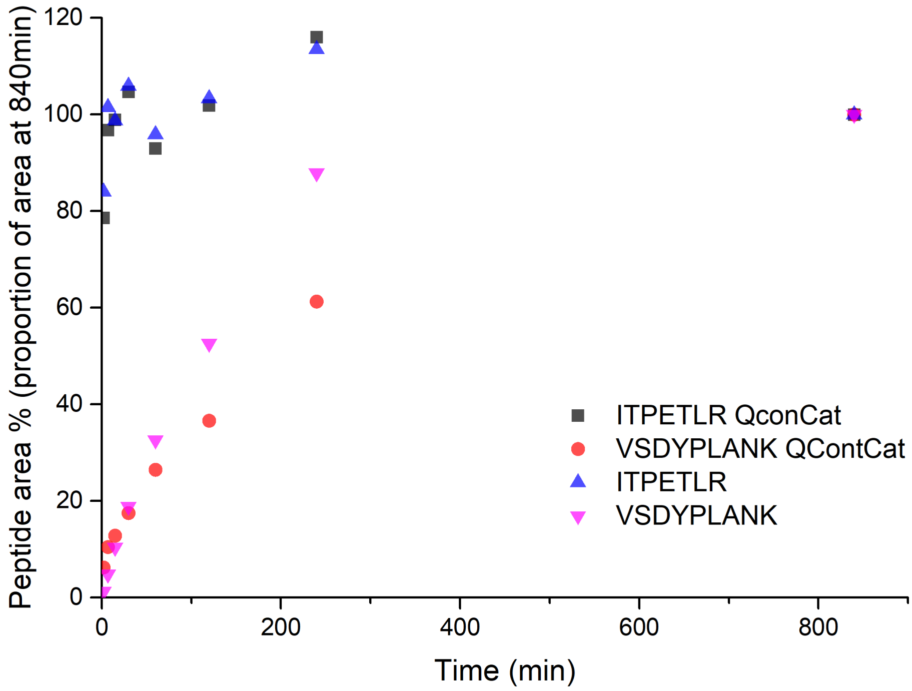

Supplementary Fig. 11. Digestion time-course analysis of target peptides of PduE. Differing digestion kinetics between the target peptide VSDYPLANK and QconCAT standard were observed. Slower digestion of QconCAT standard of this peptide could have led to over-estimation of the amount of PduE, thus the absolute quantification value from the peptide VSDYPLANK was excluded.

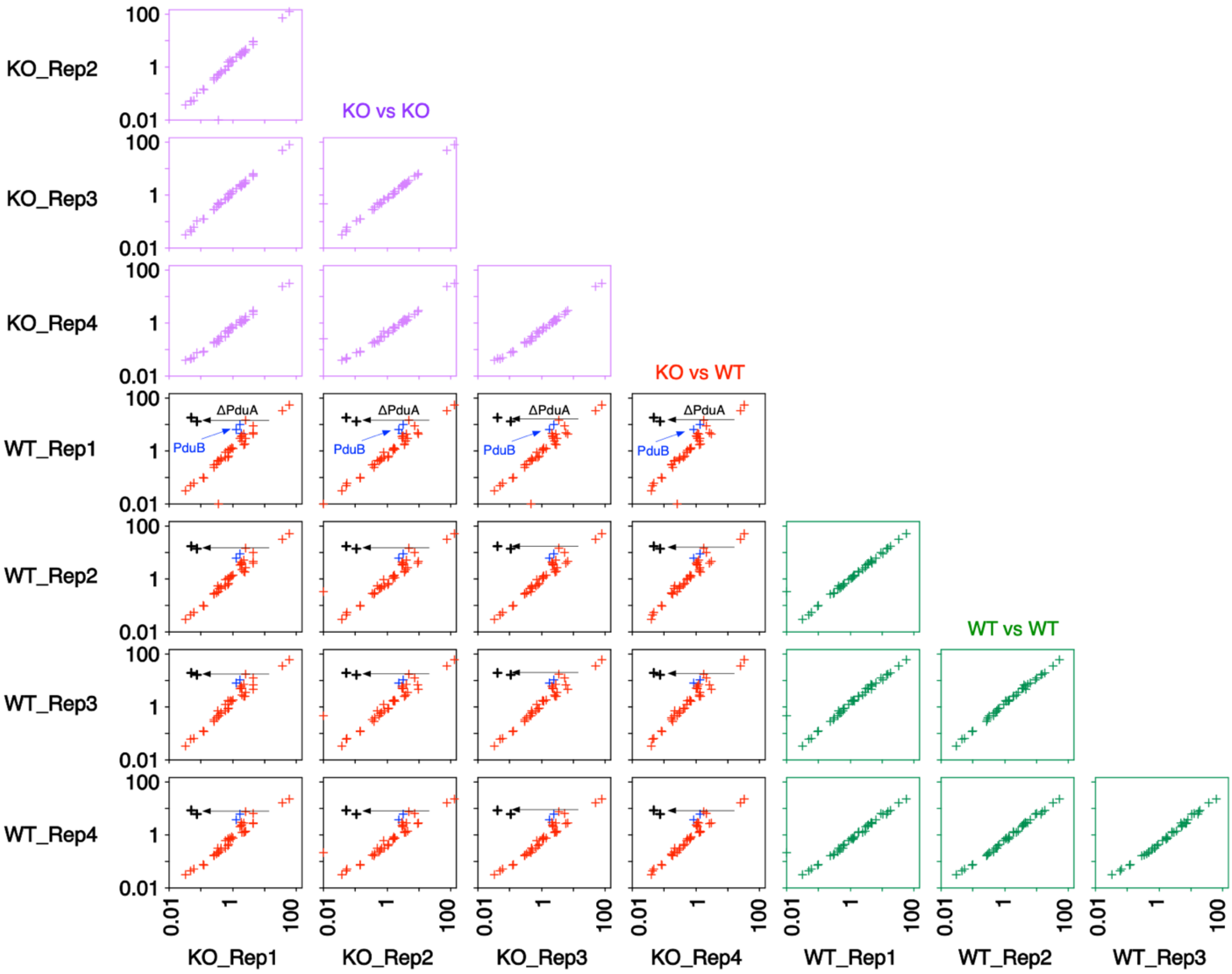

Supplementary Fig. 12. Comparative quantification analysis across all biological replicates. For each WT or Δ*pduA*-Pdu MCP (KO) sample (4 of each), the absolute quantification values (log 10 scale, four cycles) were plotted in a comparative matrix. The internal comparisons of WT (green) or KO (purple) indicate the overall experimental consistency of the study. The comparisons of WT and KO (central 4 by 4 block, red) indicate that most protein levels are essentially unchanged. Most notable is the reduction of PduA (black crosses, the target of the KO) which exhibits a high level of reduction – the non-zero value probably reflects the level of chemical noise in the quantification (about 0.1%). There was a small but significant degree of reduction in PduB (blue crosses) seen consistently across all replicates.

Supplementary Note 1: MS1-based QconCAT analysis was chosen as the precise method for absolute protein quantification of Pdu MCPs

The QconCAT method was invented by Beynon et al. as a method to provide multiplexed absolute peptide standards for a set of target proteins in isotope dilution standard experiments [6]. Since then, QconCAT constructs have been used for the quantification of any peptide that is reproducibly produced by cleavage from any sample [7]. The versatility of the QconCAT strategy has been demonstrated by a wide range of research studies, to study the stoichiometry of protein complexes [8,9], measure clinically relevant proteins in Alzheimer's disease [10], quantify posttranslational modifications [11,12], distinguish and quantify protein isoforms [13], and interrogate the mammalian host response to pathogens [14]. Recently, Beynon et al. reported the expression of QconCAT peptides in a robust, cell-free system [15]. The new expression approach, named MEERCAT (Multiplexed Efficient Expression of Recombinant QconCATs), rescues QconCATs that previously were unable to be expressed in bacteria and can reduce the incidence of proteolytic damage to QconCATs.

This study stands on the QconCAT and MEERCAT methods to quantify a total of 22 proteins within the PDU microcompartments using a synthetic QconCAT peptide that was cell-free expressed. The studying samples were purified protein complexes (not a whole proteome) and thus, had a very simple LC-MS profile, and because we had an accurately made multiplexed standard, our strategy for quantification was both robust and accurate. Additionally, whilst we used absolute quantification to avoid the known issues of label-free quantification, in which the response factor for different peptides is inconsistent, the primary goal of this study was to establish protein stoichiometry. The key factor therefore was to calculate the ratios of the different subunits as these ratios are actually independent of the quantity of the standard QconCAT.

Most global proteomic analyses combine peptide identification and quantification into a single experiment. Frequently this means that the instrument’s quantification performance is compromised in order to allow the machine to acquire the scans required for identification. In targeted proteomics, where the focus is to obtain the highest quality quantification data, it is routine to decouple the identification from quantification and perform them in separate analytical runs so the instrument can be used in optimum settings for quantification.

For comparison, in the data-dependent acquisition (DDA) method, the mass spectrometer switches between MS1 scans for quantification and MS2 scans for identification. The machine spends much longer acquiring MS2 spectra than MS1 spectra and so the chromatographic peak is sampled at lower frequency producing less accurate data. In terms of precision and accuracy, an MS1 only approach is superior because the machine is constantly sampling the chromatographic output. In targeted quantification, the peak identities have been determined based on previous identification runs using the same instrument platform, but using a DDA method with database search. The peaks of interest are therefore defined by m/z and by elution time.

Parallel reaction monitoring (PRM) is an important technique that allows mass spectrometers designed for global approaches to produce data with similar sensitivity to dedicated targeted instrumentation. In PRM, the quadrupole is cycled through different isolation values corresponding to the peptides of interest. This means that it is a scanning process and the duty cycle of the instrument drops which reduces sensitivity, precision and accuracy. As an MS2 based method, it does have much greater selectivity than MS1 methods, and on complex samples, this increased selectivity can become more important than sensitivity and the method produces high quality data. The identification runs on our samples indicated that they were of very low complexity, approximately 50 – 60 proteins (~ 500 peptides) dependant on sample. On such a simple sample, the increased selectivity of PRM is not warranted when compared to the cost of a drop in duty cycle. We applied the PRM method on a single sample, and our PRM test run produced very similar data compared to our MS1 approach. Although the PRM data were of high quality, because PRM is a scanning technique there were cases where the sampling frequency was lower with the PRM method than

with the high resolution MS1 method, despite employing scheduled isolations. This reduction of sampling frequency combined with the simplicity of sample were compelling factors in our choice of a high resolution MS1 method for quantification.

Although presented as a straight MS1 quantification, the QconCATs quantification is determined by extracting the peak areas of the first three isotope peaks of the peptide *m/z* envelope. In the absence of contamination, the intensity pattern of these areas should match the theoretical isotope distribution of the peptide and the patterns of analyte and standard should be similar. The software used to produce the quantitative data, Skyline, produces dot product metrics for both of these measures, idotp for the relationship between theoretical and experimental and rdotp for the relationship between analyte and standard. For all peptides the idotp values are above 0.8 and the rdotp was above 0.95. These high dotp scores and clean extractions also indicate that there was negligible interference in the ion signals extracted for quantification.

We used parameters optimised solely for quantification, operating the instrument at maximum resolution (fwhm 240,000 at 200 *m/z*, far higher than any DDA or parallel reaction monitoring experiment) and high sampling speed (0.6 sec). At these parameter values the mass window extracted for quantification are exceptionally narrow (approximately 7 ppm). The use of ^{13}C labelled internal standards means that the retention time and entire elution profile of the analyte and standard peak are identical. The high sampling speed means that elution profile is accurately defined allowing confidence in peak assignment.

The QConCAT has been analysed and is known not to contain post-translational modifications. This means that a single peptide is sufficient for quantification. In a previous large scale QConCAT study [4], we compared quantification of QConCATS by SDS-PAGE densitometry, GluFib MS and by amino acid hydrolysis, the normal gold standard of protein quantification. This comparison verified the accuracy of GluFib quantification of QConCATS. Adding further quantitative peptides to the QConCAT would be possible, but in order to quantify the QConCAT, the partner accurately quantified pure peptide sequence would also be required. The GluFib sequence was chosen for QConCAT quantification because there is a commercially available accurately quantified peptide.

Supplementary References:

1. Zinder ND, Lederberg J. Genetic exchange in Salmonella. *J Bacteriol* **64**, 679 (1952).
2. Datsenko KA, Wanner BL. One-step inactivation of chromosomal genes in Escherichia coli K-12 using PCR products. *Proc. Natl. Acad. Sci. U.S.A* **97**, 6640 (2000).
3. Koskiniemi S, Pränting M, Gullberg E, Näsvall J, Andersson DI. Activation of cryptic aminoglycoside resistance in Salmonella enterica. *Mol Microbiol* **80**, 1464-1478 (2011).
4. Lawless C*, et al.* Direct and absolute quantification of over 1800 yeast proteins via selected reaction monitoring. *Mol Cell Proteomics*, mcp. M115. 054288 (2016).
5. Faulkner M*, et al.* Direct characterization of the native structure and mechanics of cyanobacterial carboxysomes. *Nanoscale* **9**, 10662-10673 (2017).
6. Beynon RJ, Doherty MK, Pratt JM, Gaskell SJ. Multiplexed absolute quantification in proteomics using artificial QCAT proteins of concatenated signature peptides. *Nat Methods* **2**, 587-589 (2005).
7. Pratt JM, Simpson DM, Doherty MK, Rivers J, Gaskell SJ, Beynon RJ. Multiplexed absolute quantification for proteomics using concatenated signature peptides encoded by QconCAT genes. *Nat Protoc* **1**, 1029-1043 (2006).
8. Al-Majdoub ZM, Carroll KM, Gaskell SJ, Barber J. Quantification of the Proteins of the Bacterial Ribosome Using QconCAT Technology. *J Proteome Res* **13**, 1211-1222 (2014).
9. Nanavati D, Gucek M, Milne JLS, Subramaniam S, Markey SP. Stoichiometry and absolute quantification of proteins with mass spectrometry using fluorescent and isotope-labeled concatenated peptide standards. *Mol Cell Proteomics* **7**, 442-447 (2008).
10. Chen JJ, Wang MY, Turko IV. Mass spectrometry quantification of clusterin in the human brain. *Mol Neurodegeneration* **7**, (2012).
11. Brownridge P*, et al.* Global absolute quantification of a proteome: Challenges in the deployment of a QconCAT strategy. *Proteomics* **11**, 2957-2970 (2011).
12. Johnson H, Eyers CE, Eyers PA, Beynon RJ, Gaskell SJ. Rigorous Determination of the Stoichiometry of Protein Phosphorylation Using Mass Spectrometry. *J. Am. Soc. Mass Spectrom* **20**, 2211-2220 (2009).
13. Chen JJ, Wang MY, Turko IV. Quantification of Amyloid Precursor Protein Isoforms Using Quantification Concatamer Internal Standard. *Analytical Chemistry* **85**, 303-307 (2013).
14. Bislev SL*, et al.* Quantotypic Properties of QconCAT Peptides Targeting Bovine Host Response to Streptococcus uberis. *J Proteome Res* **11**, 1832-1843 (2012).
15. Takemori N*, et al.* MEERCAT: Multiplexed Efficient Cell Free Expression of Recombinant QconCATs For Large Scale Absolute Proteome Quantification. *Mol Cell Proteomics* **16**, 2169-2183 (2017).
