# Supplementary material for: Decoding the stoichiometric composition and organisation of bacterial metabolosomes
Source: Nat Commun. 2020 Apr 24;11:1976. doi: 10.1038/s41467-020-15888-4 (PMC7181861; doi:10.1038/s41467-020-15888-4)
Supplement: Supplementary file 2 — Reporting Summary [file 41467_2020_15888_MOESM2_ESM.pdf]

nature research

Corresponding author(s): Professor Luning Liu, NCOMMS-19-26566

Last updated by author(s): Mar 14, 2020

Reporting Summary

Nature Research wishes to improve the reproducibility of the work that we publish. This form provides structure for consistency and transparency in reporting. For further information on Nature Research policies, see Authors & Referees and the Editorial Policy Checklist .

Statistics

For all statistical analyses, confirm that the following items are present in the figure legend, table legend, main text, or Methods section.

n/a	Confirmed	
☐	☒	The exact sample size (n) for each experimental group/condition, given as a discrete number and unit of measurement
☐	☒	A statement on whether measurements were taken from distinct samples or whether the same sample was measured repeatedly
☐	☒	The statistical test(s) used AND whether they are one- or two-sided<br>*Only common tests should be described solely by name; describe more complex techniques in the Methods section.*
☒	☐	A description of all covariates tested
☐	☒	A description of any assumptions or corrections, such as tests of normality and adjustment for multiple comparisons
☐	☒	A full description of the statistical parameters including central tendency (e.g. means) or other basic estimates (e.g. regression coefficient) AND variation (e.g. standard deviation) or associated estimates of uncertainty (e.g. confidence intervals)
☐	☒	For null hypothesis testing, the test statistic (e.g. F, t, r) with confidence intervals, effect sizes, degrees of freedom and P value noted<br>*Give P values as exact values whenever suitable.*
☒	☐	For Bayesian analysis, information on the choice of priors and Markov chain Monte Carlo settings
☒	☐	For hierarchical and complex designs, identification of the appropriate level for tests and full reporting of outcomes
☒	☐	Estimates of effect sizes (e.g. Cohen's d, Pearson's r), indicating how they were calculated

Our web collection on statistics for biologists contains articles on many of the points above.

Software and code

Policy information about availability of computer code

Data collection	Transmission electron microscopy (TEM) data collected by SIS Megaview III camera. Liquid chromatography–mass spectrometry (LC-MS) were conducted on a QExactive HF quadrupole-Orbitrap mass spectrometer coupled to a Dionex Ultimate 3000 RSLC nano-liquid chromatograph (Thermo Fisher, UK, Xcalibur software version 4.1). Confocal microscopy LSM780 used ZEISS ZEN microscope software to run the confocal and collect images.
Data analysis	TEM and confocal microscopy data was analyzed with ImageJ-win64. Student’s t-test conducted by IBM SPSS Statistics 24. Proteomic analysis used Skyline (64 bit) version 4.1.0.18169 open-source Windows client application from the MaCoss Lab, Department of Genome Sciences, University of Washington. An in-house Mascot Server search engine (version 2.6.2), www.matrixscience.com, was used to search protein databases.

For manuscripts utilizing custom algorithms or software that are central to the research but not yet described in published literature, software must be made available to editors/reviewers. We strongly encourage code deposition in a community repository (e.g. GitHub). See the Nature Research guidelines for submitting code & software for further information.

Data

Policy information about availability of data

All manuscripts must include a data availability statement. This statement should provide the following information, where applicable:

- Accession codes, unique identifiers, or web links for publicly available datasets
- A list of figures that have associated raw data
- A description of any restrictions on data availability

The mass spectrometry proteomics data associated with Supplementary Figs. 6 and 11 have been deposited to the ProteomeXchange Consortium via the PRIDE partner repository with the dataset identifier PXD015111 and 10.6019/PXD015111.
Reviewer account details: Username: reviewer01037@ebi.ac.uk Password: hc9rtj4O
The source data underlying Table1, Figs 1e, 3a–b, 4f, 4i and Supplementary Figs. 2, 3a-b and 10 are provided as a Source Data file. All data are available from the

corresponding author upon request.
Uniprot database was used to search the unique of the target peptides of each Pdu protein.

Field-specific reporting

Please select the one below that is the best fit for your research. If you are not sure, read the appropriate sections before making your selection.

- [x] Life sciences
- [ ] Behavioural & social sciences
- [ ] Ecological, evolutionary & environmental sciences

For a reference copy of the document with all sections, see nature.com/documents/nr-reporting-summary-flat.pdf

Life sciences study design

All studies must disclose on these points even when the disclosure is negative.

Sample size	The sample size for TEM data were determined by the number of available images which were of high quality and sufficient to make accurate measurements. Four biological replicates were analyzed by LC-MS, which is sufficient for protein quantification.
Data exclusions	For TEM data, only particles with clear boundaries were used for accurate measurements. Representative high quality confocal images were shown in the paper. Low quality and low resolution images were excluded, which is standard practice for TEM and confocal imaging and can be considered as pre-established criteria. No proteomics data were excluded except one peptide for PduE, due to differing digestion kinetics between the target peptide and QconCAT standard.
Replication	All of the experiments were repeated more than three times, and were reproduced successfully.
Randomization	Bacteria colonies were selected randomly for cell culture, purification, confocal imaging and proteomic analysis. Purified particles were selected randomly for TEM imaging. LC-MS acquisition was carried out on a randomised sample order.
Blinding	Blinding was not possible because sample preparation and data collection were conducted by the same investigators.

Reporting for specific materials, systems and methods

We require information from authors about some types of materials, experimental systems and methods used in many studies. Here, indicate whether each material, system or method listed is relevant to your study. If you are not sure if a list item applies to your research, read the appropriate section before selecting a response.

Materials & experimental systems

n/a	Involved in the study
[x]	[ ] Antibodies
[x]	[ ] Eukaryotic cell lines
[x]	[ ] Palaeontology
[x]	[ ] Animals and other organisms
[x]	[ ] Human research participants
[x]	[ ] Clinical data

Methods

n/a	Involved in the study
[x]	[ ] ChIP-seq
[x]	[ ] Flow cytometry
[x]	[ ] MRI-based neuroimaging
